# The *Medicago truncatula* hydrolase MtCHIT5b degrades Nod factors of *Sinorhizobium meliloti* and cooperates with MtNFH1 to regulate the nodule symbiosis

**DOI:** 10.3389/fpls.2022.1034230

**Published:** 2022-11-17

**Authors:** Ru-Jie Li, Chun-Xiao Zhang, Sheng-Yao Fan, Yi-Han Wang, Jiangqi Wen, Kirankumar S. Mysore, Zhi-Ping Xie, Christian Staehelin

**Affiliations:** ^1^ State Key Laboratory of Biocontrol, School of Life Sciences, Sun Yat-sen University, Guangzhou, China; ^2^ Guangdong Provincial Key Laboratory of Plant Resources, School of Life Sciences, Sun Yat-sen University, Guangzhou, China; ^3^ Institute for Agricultural Biosciences, Oklahoma State University, Ardmore, OK, United States

**Keywords:** *Medicago truncatula*, *Sinorhizobium meliloti*, Nod factors, Nod factor hydrolysis, symbiosis

## Abstract

Nod factors secreted by nitrogen-fixing rhizobia are lipo-chitooligosaccharidic signals required for establishment of the nodule symbiosis with legumes. In *Medicago truncatula*, the Nod factor hydrolase 1 (MtNFH1) was found to cleave Nod factors of *Sinorhizobium meliloti*. Here, we report that the class V chitinase MtCHIT5b of *M. truncatula* expressed in *Escherichia coli* can release lipodisaccharides from Nod factors. Analysis of *M. truncatula* mutant plants indicated that MtCHIT5b, together with MtNFH1, degrades *S. meliloti* Nod factors in the rhizosphere. *MtCHIT5b* expression was induced by treatment of roots with purified Nod factors or inoculation with rhizobia. MtCHIT5b with a fluorescent tag was detected in the infection pocket of root hairs. Nodulation of a *MtCHIT5b* knockout mutant was not significantly altered whereas overexpression of *MtCHIT5b* resulted in fewer nodules. Reduced nodulation was observed when *MtCHIT5b* and *MtNFH1* were simultaneously silenced in RNA interference experiments. Overall, this study shows that nodule formation of *M. truncatula* is regulated by a second Nod factor cleaving hydrolase in addition to MtNFH1.

## Introduction

Legumes (*Fabaceae*) can form a mutualistic relationship with nitrogen-fixing bacteria, called rhizobia. In formed nodules, carbon and nutrients are provided by host plants, while rhizobia differentiated into bacteroids deliver fixed nitrogen to the plant. Formation of nodules on legume roots depends on a molecular signal exchange between both symbiotic partners (Perret et al., 2000; Mergaert et al., 2020). Specific flavonoids or isoflavonoids are secreted by legume roots and interact with rhizobial NodD proteins to induce expression of bacterial nodulation (*nod)* genes (Liu and Murray, 2016). The *nod* genes are responsible for biosynthesis and secretion of Nod factors. These rhizobial signal molecules are lipo-chitooligosaccharides (modified chitin oligosaccharides, LCOs) containing β-1,4-linked *N*-acetylglucosamine residues with three to five sugars and a fatty acid chain at the non-reducing end. Furthermore, Nod factors with additional modifications such as a sulfate group at the reducing end are produced by many strains (Perret et al., 2000). *Sinorhizobium meliloti* strains overexpressing certain *nod* genes can secrete high amounts of 6-*O*-sulfated Nod factors with a C16:2 fatty acid (Lerouge et al., 1990; Schultze et al., 1992). Nod factor production is a common feature among almost all rhizobia except certain *Bradyrhizobium* strains (Giraud et al., 2007).

Nod factor signaling in host legumes triggers expression of symbiotic genes required for bacterial infection and nodule development. Nod factor receptor genes have been first identified in the model legumes *Lotus japonicus* and *Medicago truncatula* (Roy et al., 2020; Yang et al., 2022). When Nod factor receptor genes of *L. japonicus* were ectopically expressed in *M. truncatula*, roots could be nodulated by *Mesorhizobium loti*, which is a natural symbiont of *L. japonicus* (Radutoiu et al., 2007). Such experiments indicated that Nod factor receptors are determinants of host specific nodulation. In response to Nod factor perception, a calcium oscillation response (calcium spiking) is induced in nuclei of host cells. Calcium- and calmodulin-dependent protein kinases are the sensors of nuclear calcium spiking (Yang et al., 2022). These protein kinases can phosphorylate transcription factors such as CYCLOPS in *L. japonicus* (Singh et al., 2014) and GmSTF3 (TGACG-Motif Binding Factor 3) in soybean (Wang et al., 2021). Ultimately, additional transcription factors such as NIN (Nodule Inception) trigger expression of symbiotic genes required for bacterial infection and formation of nodule primordia (Roy et al., 2020; Yang et al., 2022).

Rhizobia often enter host plants *via* root hairs. Root hair curling results in formation of an infection pocket in which the bacteria form a microcolony (Fournier et al., 2015). Following endocytosis, infection threads are initiated which later elongate in the root hairs until they reach the cortical cells where they branch and colonize the formed nodule primordium. The formation of infection pockets is accompanied by secretion of proteins into the symbiotic interface. In *M. truncatula* for example, MtENOD11 (Early Nodulin 11), a proline-rich cell wall protein, has been reported to be accumulated within the infection pocket (Charron et al., 2004; Fournier et al., 2015). *MtENOD11* is a suitable marker gene for studying activation of Nod factor signaling in *M. truncatula* roots (Charron et al., 2004).

Chitinases are enzymes that hydrolyze β-1,4 linkages in chitin (poly *N*-acetylglucosamine). Plant chitinases belong to the glycoside hydrolase families 18 (class III and V chitinases) and 19 (class I, II and IV chitinases) (Drula et al., 2022). Since Nod factors are also composed of β-1,4-linked *N*-acetylglucosamine, certain plant chitinases can cleave Nod factors thereby forming a hydrophobic cleavage product with an acyl chain and a hydrophilic carbohydrate moiety. Different modifications in Nod factors can render certain Nod factors partially or completely resistant to hydrolysis by plant chitinases (Staehelin et al., 1994a; Staehelin et al., 1994b; Goormachtig et al., 1998; Schultze et al., 1998; Ovtsyna et al., 2000; Tian et al., 2013; Zhang et al., 2016). Purified Nod factors applied to the rhizosphere of legumes were rapidly degraded and inactivated by plant hydrolases within few hours (Staehelin et al., 1994b; Heidstra et al., 1994; Staehelin et al., 1995; Ovtsyna et al., 2000; Ovtsyna et al., 2005; Tian et al., 2013; Cai et al., 2018).

Nod factor signaling induces expression of genes encoding Nod factor cleaving enzymes. In *Sesbania rostrata*, expression of a Nod factor cleaving class III chitinase gene was found to be induced by applied Nod factors and when roots were inoculated with *Azorhizobium caulinodans* (Goormachtig et al., 1998). In *M. truncatula*, expression of a class V chitinase gene was found to be up-regulated by inoculation with *S. meliloti* bacteria and Nod factor treatments (Salzer et al., 2004). Enzyme tests with recombinant protein expressed in *E. coli* showed that the enzyme lacks chitin-cleaving activity but can efficiently hydrolyze Nod factors. The protein was therefore named MtNFH1 (Nod factor hydrolase 1; Tian et al., 2013). MtNFH1 cleaves tetrasaccharidic and pentasaccharidic Nod factors of *S. meliloti*, thereby forming lipodisaccharides. Based on the known structures of the class V chitinases of tobacco (*Nicotiana tabacum*) and *Arabidopsis thaliana*, a homology modeling and substrate docking simulation was performed for MtNFH1. The results suggested a binding cleft for the Nod factor substrate that specifically accommodates the fatty acid chain (Tian et al., 2013).

Recently, a *MtNFH1* knockout mutant was found to show delayed root hair infection, suggesting that too high Nod factor concentrations impair the formation of an infection thread. Furthermore, *MtNFH1* mutant plants exhibited abnormal nodule branching at 20 days post inoculation (dpi). These results suggested that spatio-temporal fine-tuning of Nod factor levels affect the nodule symbiosis at early and late symbiotic stages (Cai et al., 2018). In *L. japonicus*, a class V chitinase gene, *LjCHIT5*, has been found to be implicated in the symbiosis with *M. loti*. *LjCHIT5* knockout mutant plants formed fewer pink (nitrogen-fixing) nodules and had a defective nodule infection phenotype. Furthermore, the mutants showed reduced infection thread elongation inside nodule primordia while root hair infection was not affected (Malolepszy et al., 2018).

The differences in *MtNFH1* and *LjCHIT5* mutant phenotypes suggest that additional Nod factor-cleaving enzymes of *M. truncatula* could play a symbiotic role. In *M. truncatula*, the class V chitinase genes *MtCHIT5b* and *MtCHIT5a* and are most related to *MtNFH1* (amino acid sequence identity of 75% and 46%, respectively). *MtCHIT5b* and *MtNFH1* are located in tandem on chromosome 4 of the *M. truncatula* genome, suggesting gene duplication during evolution. Recombinant MtCHIT5a and MtCHIT5b proteins expressed in *Escherichia coli* can hydrolyze chitooligosaccharides but Nod factor cleaving activity has not yet been demonstrated for these proteins (Tian et al., 2013; Zhang et al., 2016). However, MtCHIT5b with a serine-to-proline substitution at the predicted substrate binding cleft was found to possess Nod factor hydrolase activity similar to MtNFH1. Conversely, a corresponding proline-to-serine substitution in MtNFH1 led to a variant that did not release detectable amounts of cleavage products from *S. meliloti* Nod factors (Zhang et al., 2016).

In this study, we found that recombinant MtCHIT5b expressed in *E. coli* could hydrolyze *S. meliloti* Nod factors under newly established purification and test conditions. *MtCHIT5b* expression in young root hairs was rapidly induced by applied Nod factors or rhizobial inoculation. Expression of MtCHIT5b with fluorescent tags in *M. truncatula* showed that the enzyme is mainly localized in the infection pocket of a curled root hair. Analysis of *MtCHIT5b* mutant plants indicated reduced Nod factor hydrolysis in the rhizosphere but no obvious symbiotic phenotype was observed. In contrast, *MtCHIT5b* overexpression negatively affected nodule formation. RNA interference experiments indicated that simultaneous silencing of *MtCHIT5b* and *MtNFH1* impairs nodule formation.

## Materials and methods

### Strains, vectors and plasmids

*Escherichia coli* DH5α and BL21 (DE3) (Novagen, Madison, USA) were grown in Luria-Bertani (LB) medium (5 g L^-1^ yeast extract, 10 g L^-1^ tryptone, 10 g L^-1^ NaCl) at 37 °C. *Sinorhizobium meliloti* Rm41 and fluorescent derivatives were cultured in TY medium (3 g L^-1^ yeast extract, 5 g L^-1^ tryptone, 0.4 g L^-1^ CaCl_2_) at 27 °C. For plant inoculation, bacteria were centrifuged (4 000 rpm for 10 min) and the pellets were resuspended in 10 mM sterilized MgSO_4_ (OD_600_ ≈ 0.2). *S. meliloti* 1021 (pEK327) was grown in defined medium and used for Nod factor purification (Schultze et al., 1992; Tian et al., 2013). *Agrobacterium rhizogenes* ARqua-1 (Quandt et al., 1993) was cultured in TY medium at 27 °C. DNA constructs used for this study were cloned into the plasmids pET-28b (Novagen, Darmstadt, Germany), pBBR1MCS-2 (Kovach et al., 1995), pUB-*GFP* (Maekawa et al., 2008), pCAMBIA1302 and pCAMBIA1305 (http://www.cambia.org/).

To obtain red fluorescent *S. meliloti* bacteria (strain Rm41-*mCherry*), DNA containing the spectinomycin resistance gene promoter of pHP45 (Quandt and Hynes, 1993) and the *mCherry* gene (Shaner et al., 2004) was cloned into pBBR1MCS-2 (Kovach et al., 1995) using *Xho*I/*Xba*I. The plasmid was then mobilized into *S. meliloti* Rm41 by tri-parental mating (Figurski and Helinski, 1979). A similar approach was used for construction of Rm41-*mTFP1* expressing mTFP1 (Ai et al., 2006).

For *MtCHIT5b* promoter-β-glucuronidase gene (*GUS*) fusion analysis, a 3 022-bp promoter sequence flanking the coding region of *MtCHIT5b* was amplified from genomic DNA of *M. truncatula* R108 and cloned into pCAMBIA1305 with *BamH*I/*Nco*I, generating a promoter-*GUS* fusion (*MtCHIT5b*p-*GUS* construct). Then, a red fluorescent protein (RFP) expression cassette (Chen et al., 2009) was cloned into the constructed binary vector using *Kpn*I.

For subcellular localization, DNA encoding MtCHIT5b fused to mCherry (MtCHIT5b:mCherry) was amplified by overlap extension PCR using genomic DNA of *M. truncatula* R108 and *mCherry* DNA as a template. The amplicon was then inserted with *Xba*I/*Kpn*I into pUB-*GFP* containing a *Ubiquitin* gene promoter from *L. japonicus*, which possesses strong promoter activity in *M. truncatula* (Lei et al., 2015; Kovács et al., 2022). Furthermore, a construct encoding MtCHIT5b fused to GFP with an upstream *MtCHIT5b* promoter sequence was used for subcellular localization. The 3 022-bp *MtCHIT5b* promoter sequence (amplified from genomic DNA of *M. truncatula* R108) was cloned into pCAMBIA1302 with *BamH*I/*Nco*I. Next, the coding region of *MtCHIT5b* was amplified from genomic DNA of *M. truncatula* R108 and the overhangs were removed with *Nco*I. The Hieff Clone™ Plus Multi One Step Cloning Kit (Yeasen, Shanghai, China) was then used to insert the *MtCHIT5b* coding sequence between the promoter sequence and the *GFP* tag. For an additional plasmid, an *RFP* expression cassette (Chen et al., 2009) was inserted into the binary vector with the help of *Kpn*I.

Binary vectors with RNAi constructs were used to silence *MtCHIT5b*/*MtNFH1* expression in *M. truncatula* roots. A DNA fragment containing a 613-bp sequence of *MtCHIT5b* fragment was amplified from genomic DNA of *M. truncatula* R108 and inserted with *EcoR*I/*Sma*I into a pBluescript vector containing a DNA spacer region. The reverse complement fragment was also amplified and inserted into the vector with *BamH*I/*Xba*I. The whole dsRNA1 cassette was then excised with *Hind*III/*Sac*I and inserted into pCAMBIA1305. Finally, an *RFP* expression cassette (Chen et al., 2009) was cloned into the *Hind*III site of the constructed vector. In addition, a similar binary vector was constructed which contains the 667-bp dsRNA2 sequence derived from *MtNFH1* (Tian et al., 2013).

To constitutively overexpress *MtCHIT5b* in roots of *M. truncatula*, the coding sequence of *MtCHIT5b* was amplified from genomic DNA of *M. truncatula* R108 and inserted into pUB-*GFP* with *Xba*I/*Kpn*I. The promoter-*MtCHIT5b* construct was then PCR amplified and inserted into pCAMBIA1305 carrying an *RFP* expression cassette with the help of the cloning kit mentioned above.

Construction of plasmids used for expression of MtCHIT5b and MtNFH1 proteins in *E. coli* has been reported previously (Tian et al., 2013; Zhang et al., 2016). Further information on strains and plasmids used in this study can be found in **Supplementary Table 1**. Primers are listed in **Supplementary Table 2**.

### Plant materials, seed germination and plant growth conditions

*Medicago truncatula* ecotype R108 and Jemalong A17 were used in this study. The *Tnt1* insertion mutant lines NF16543 (*chit5b*) and NF11260 (*nfh1-3b*) were obtained from the *M. truncatula Tnt1* mutant collection currently hosted at the Institute of Agricultural Biosciences, Oklahoma State University, Ardmore, USA (Tadege et al., 2008; Pislariu et al., 2012). In contrast to the previously described *nfh1-3* mutant (Cai et al., 2018), *nfh1-3b* inoculated with *S. meliloti* did not form branched nodules at 3 weeks post inoculation (wpi) under the experimental conditions used in this study. The nodulation signaling mutant *dmi3-1* of ecotype Jemalong A17 (TRV25; Sagan et al., 1998) was kindly provided by Dr. Clare Gough (INRAE-CNRS, Castanet-Tolosan, France).

*M. truncatula* seeds were scarified with sandpaper, sterilized in 10% commercial bleach for 5 min and finally rinsed in sterile water for several times. The seeds were then placed on inverted 1.2% (v/v) water agar plates in the dark at 4 °C for 48 h and later at 23 ± 1 °C for 18 h. Unless otherwise specified, seedlings were grown on 1% (v/v) agar plates containing BNM (buffered nodulation medium) (Ehrhardt et al., 1992). The lower part of the plate was covered with aluminum foil. The plates were then placed in a growth room at an angle of ~60°. Special growth conditions were established for root transformation and nodulation experiments. All plants were kept in an air-conditioned growth room at 23 ± 1 °C under 16/8-h light/dark conditions as described previously (Cai et al., 2018).

### Transformation of *M. truncatula* roots

Binary vectors were transformed into competent *A. rhizogenes* ARqua-1 by electroporation (1 800 V for 5 ms). Cells were sprayed on TY plates containing 50 mg L^-1^ kanamycin and 50 mg L^-1^ streptomycin and incubated at 27 °C. Formed colonies were confirmed by PCR. Hairy root transformation was performed according to the protocol described by Boisson-Dernier et al. (2001) with minor modifications. At the stage of emerging hairy roots, seedlings were transferred to solid HRE plates (Díaz et al., 2005) supplemented with 300 μg/mL timentin and kept under growth conditions as reported (Cai et al., 2018). Ten days later, seedlings were analyzed by fluorescence microscopy and non-transgenic roots lacking RFP fluorescence were removed. A similar transformation efficiency was observed for the different binary vectors used in this study.

### Genotyping of *Tnt1* insertion mutant lines

Genomic DNA was isolated from young leaves of *M. truncatula* (R108 wild-type plants and mutant lines) using cetyltrimethylammonium bromide. To confirm the exact *Tnt1* insertion sites in mutant plants, the isolated DNA was used as template for PCRs using primers designed from the flanking regions of the *Tnt1* insertion sites. Information on used primers is provided in **Supplementary Table 2**.

### Purification of Nod factors and HPLC analysis

Purification of Nod factors from *S. meliloti* 1021 (pEK327) was performed as described previously (Staehelin et al., 1994b; Tian et al., 2013) with the exception that Amberlite XAD-2 resin (20-60 mesh; Supelco, Merck, Kenilworth, USA) instead of *n-*butanol was used for the initial purification step with bacterial culture supernatants. For purification of NodSm-IV(C16:2, S) and NodSm-V(C16:2, S), the culture supernatant was adjusted to pH 9.5 and shaken at 27 °C for 24 h. Amberlite XAD-2 resin was extensively washed with acetone, then with methanol and final equilibrated with water. Subsequently, 40 g of the conditioned resin was added to 1 L of the prepared bacterial culture supernatant. After incubation overnight (27 °C; 150 rpm), the resin material was collected with a metal mesh and extensively washed with water. Using a vacuum filtration system, the Nod factors were eluted from the resin with 40 mL methanol and then with 30 mL acetone. The filtrates were collected and dried in a rotavapor system. Nod factors were then fractionated by reverse-phase HPLC using a Nova Pak C18 column (3.9 x 150 mm, particle size 4 µm; Waters, Milford, USA) and 30% or 35% (v/v) acetonitrile/water containing 40 mM ammonium acetate. Fractions containing Nod factors were collected and dried using a speed-vac concentrator. C18 Sep-Pak cartridges (Waters, Milford, USA) were used for desalting purified Nod factors. Briefly, purified Nod factors were dissolved in 5 mL water containing 0.5% (v/v) dimethyl sulfoxide (DMSO) and then loaded onto the cartridges which were washed with methanol and conditioned with water. After washing with 10 mL water, the Nod factors were eluted with 5 mL methanol. The desalted Nod factors were dried in a speed-vac concentrator and re-dissolved in 1 mL water containing 0.5% (v/v) DMSO.

### Purification of recombinant MtCHIT5b

*E. coli* strain BL21 (DE3) carrying pET-28b containing *MtCHIT5b* without predicted signal peptide (Zhang et al., 2016) was used to obtain enzymatically active MtCHIT5b with an N-terminal His-tag. A single colony was cultured in 3 mL of liquid LB supplemented with 50 μg mL^-1^ kanamycin on a shaker (200 rpm at 37 °C). After incubation (12 h), 2 mL of the obtained preculture were used for inoculation of 400 mL LB (containing 50 μg mL^-1^ kanamycin) and the bacteria were cultured under the same growth conditions. When the optical density (OD_600_) reached 0.5, isopropyl-β-D-thiogalactopyranoside was added to obtain a final concentration of 0.4 mM. The cultures were then kept on the shaker at 18 °C for 20 h. Finally, the cultures were centrifuged (4 000 rpm at 4 °C for 15 min) and the bacterial pellets were collected. MtCHIT5b was purified under native conditions. Briefly, each bacterial pellet was resuspended in 8 mL lysis buffer (50 mM NaH_2_PO_4_, 300 mM NaCl, 10 mM imidazole, pH 7.5) supplemented with 1 mg/mL lysozyme. Next, the mixture was sonicated on ice with an ultrasonic processor (Ningbo Scientz Biotechnology Co., Ningbo, China; 7 s burst followed by 53 s cooling step for 18 min, 60 W). After sonication, the suspension was gently shaken at 100 rpm for 30 min and then subjected to centrifugation (12 000 rpm at 4 °C for 20 min). The clear supernatant was collected and mixed with 0.2 mL Ni-NTA resin beads (TransGen Biotech, Beijing, China), which were conditioned by washing with lysis buffer (4 °C). After incubation (100 rpm at 4 °C for 45 min), the beads were placed into a column and washed with 16 mL wash buffer (50 mM NaH_2_PO_4_, 300 mM NaCl, 20 mM imidazole, pH 7.5). Finally, MtCHIT5b was eluted from the beads with 0.3 mL elution buffer (50 mM NaH_2_PO_4_, 300 mM NaCl, 600 mM imidazole, adjusted to pH 7.5). The elution fraction was immediately mixed with an equal volume of glycerol and a 0.47-fold volume of 500 mM sodium acetate buffer (pH 4.0) to obtain a final pH of 7.0.

### Protein electrophoresis and Western blot analysis

Proteins were analyzed by 12% sodium dodecyl sulfate polyacrylamide gel electrophoresis (SDS-PAGE). Protein samples were supplemented with loading buffer (TransGen Biotech, Beijing, China), followed by boiling at 100 °C for 10 min. After electrophoresis, gels were stained with Commassie Brilliant Blue R-250. Photos were taken with a digital camera (Nikon, Nikon, Japan). For protein quantification, different concentrations of bovine serum albumin (BSA) were used as standards. A standard curve was drawn using corresponding signal intensities of protein bands as quantified by ImageJ software (NIH, Bethesda, USA). Western blot analysis was performed with nitrocellulose membranes temporarily stained with Ponceau S dye to visualize transferred proteins. The membranes were incubated with an anti-His antibody (1:10 000 dilution; TransGen Biotech, Beijing, China). A goat-anti-mouse IgG antibody coupled to horseradish peroxidase (1:10 000 dilution; TransGen Biotech, Beijing, China) served as second antibody. The membranes were developed with an enhanced chemiluminescence kit (FDbio Science, Hangzhou, China).

### Enzyme assays with Nod factors

If not otherwise specified, enzyme assays with Nod factors and test proteins were performed in 25 mM potassium phosphate buffer (pH 7.0) containing 0.5% DMSO and 10 μg mL^-1^ BSA at 37 °C. Typically, 3 nmol of a purified Nod factor substrate (NodSm-IV(C16:2, S), NodSm-V(C16:2, S) or NodSm-IV(C16:2, Ac, S)) was incubated with a 0.25 μg mL^-1^ MtCHIT5b sample (pH 7.0) in a given reaction volume. After incubation for different times (37 °C), samples were extracted by an equal volume of *n*-butanol and the collected butanol phase was dried using a speed-vac concentrator. The dried samples were resuspended in 1.2 μL DMSO and finally analyzed by HPLC using a Nova Pak C18 column and 35% (v/v) acetonitrile/water containing 40 mM ammonium acetate as mobile phase (Staehelin et al., 1994b). The chemical structures of obtained acylated cleavage products, the lipodisaccharide II(C16:2) and the *O*-acetylated lipodisaccharide II(C16:2, Ac), were confirmed by MALDI-TOF mass spectrometry. The instrument (Bruker, Bremen, Germany) was operating in the positive-ion mode and 2,5-dihydroxybenzoic acid was used as matrix. The *K*_m_ and V_max_ values of MtCHIT5b for the Nod factor substrates were determined using GraphPad Software (Dotmatics, Boston, USA). The velocity values were calculated from reactions with no more than 25% cleavage product formation.

### Treatment of plants with Nod factors

Five-day old *M. truncatula* seedlings or plants with transformed roots were placed into glass plates (6 cm in diameter) filled with 7 mL of liquid BNM containing purified Nod factors and 0.5% (v/v) DMSO. Control plants were treated with BNM containing 0.5% DMSO. After incubation for 6 or 24 h in the dark (23 ± 1 °C), plants were used for real-time quantitative PCR (RT-qPCR) analysis or subjected to GUS staining.

### Gene expression analysis by RT-qPCR

Total RNA of plant material was extracted using the Plant Total RNA Purification Kit (GeneMark, Taiwan, China) followed by cDNA synthesis with HiScript II Q RT SuperMix (Vazyme, Nanjing, China). RT-qPCR was performed with a LightCycler 480 instrument using the LightCycler 480 SYBR Green I Master Reaction Mix (Roche, Shanghai, China). The reaction conditions were as follow: (1) 95 °C for 5 min; (2) 45 cycles: 95 °C for 20 s, 55 °C for 20 s, 72 °C for 40 s; (3) 95 °C for 5 s, 60 °C for 60 s and 95 °C for 10 s. RNAs were extracted in triplicate (three biological replicates) and each corresponding cDNA was amplified three times (three technical replicates). *Medicago elongation factor* was used as a reference gene. The Ct values were obtained with the help of the LightCycler 480 software. Primers used for RT-qPCR are shown in **Supplementary Table 2**.

### GUS staining

Transgenic roots transformed with the *MtCHIT5b*p-*GUS* construct (or nodules obtained from inoculation with *S. meliloti* Rm41) were immersed in a solution (pH 7.0) containing 100 mM NaH_2_PO_4_, 10 mM EDTA disodium salt, 0.5 mM K_3_Fe(CN)_6_, 0.5 mM K_4_Fe(CN)_6_, 0.1% Triton-X100 and 0.5 mg mL^-1^ 5-bromo-4-chloro-3-indoxyl-β-D-glucuronic acid cyclohexylammonium salt (X-Gluc). The samples were exposed to a vacuum for 25 min and then incubated at 37 °C for different periods. Finally, the stained tissue was analyzed with a Nikon Eclipse Ni-E microscope. For sectioning of nodules, GUS-stained samples were fixed in 1.25% glutaraldehyde phosphate buffer (pH 5.7) under a vacuum for 1 h. Samples were embedded in 4% (w/v) agrose and 70-μm sections were obtained using a LeicaVT1200S vibrating blade microtome.

### Subcellular localization of MtCHIT5b

For subcellular localization of MtCHIT5b, the protein with a fluorescent tag was expressed in *M. truncatula* roots with the help of *A. rhizogenes*. The transformed roots were inoculated with Rm41, Rm41-*mCherry* or Rm41-*mTFP1* (OD_600_ ≈ 0.2). Fluorescence in infected root hairs was observed using a Zeiss ImagerZ1 fluorescence microscope with filters as recommended by the supplier (Carl Zeiss AG, Oberkochen, Germany). Furthermore, subcellular localization of MtCHIT5b was predicted by using the ProtComp 9.0 server (http://www.softberry.com/berry.phtml).

### Hydrolysis of Nod factors by *M. truncatula* seedlings

Five-day-old *M. truncatula* seedlings grown on BNM agar plates were transferred to 1-mL plastic syringes (Li et al., 2019). The syringes were filled with 300 μL BNM containing 0.5% (v/v) DMSO and 0.5 μM NodSm-IV(C16:2, S) to activate Nod factor signaling. After incubation in the dark (23 ± 1 °C for 18 h), the seedlings were transferred to new 1-mL syringes filled with 900 μL BNM containing 0.5% (v/v) DMSO and 5.4 μM NodSm-IV(C16:2, S). After incubation for 2 h, the seedlings were removed and the Nod factor substrate and formed II(C16:2) were extracted with an equal volume of *n*-butanol. Dried materials from three seedlings were combined to obtain a sample for HPLC analysis. In total, three HPLC samples per treatment were analyzed.

### Nodulation tests

Germinated *M. truncatula* seedlings and plants with transformed roots were transferred to sterile 300-mL plastic jar units. The upper jar contained vermiculite/expanded clay at a 3:1 (v/v) ratio and the lower jar contained sterilized B&D nutrient solution (Broughton and Dilworth, 1971) supplemented with 1 mM KNO_3_. Each jar unit contained a single plant. After 7 days, plants were inoculated with 2 mL of a rhizobial suspension (OD_600_ ≈ 0.2) or mock-inoculated with 2 mL of 10 mM sterilized MgSO_4_. The substrate of the upper jar units was then covered with sterilized quartz stones. At the time of harvest, the nodules were counted and the nodule biomass was determined for each plant.

## Results

### *MtCHIT5b* encodes a Nod factor-cleaving enzyme

**Figure 1:**
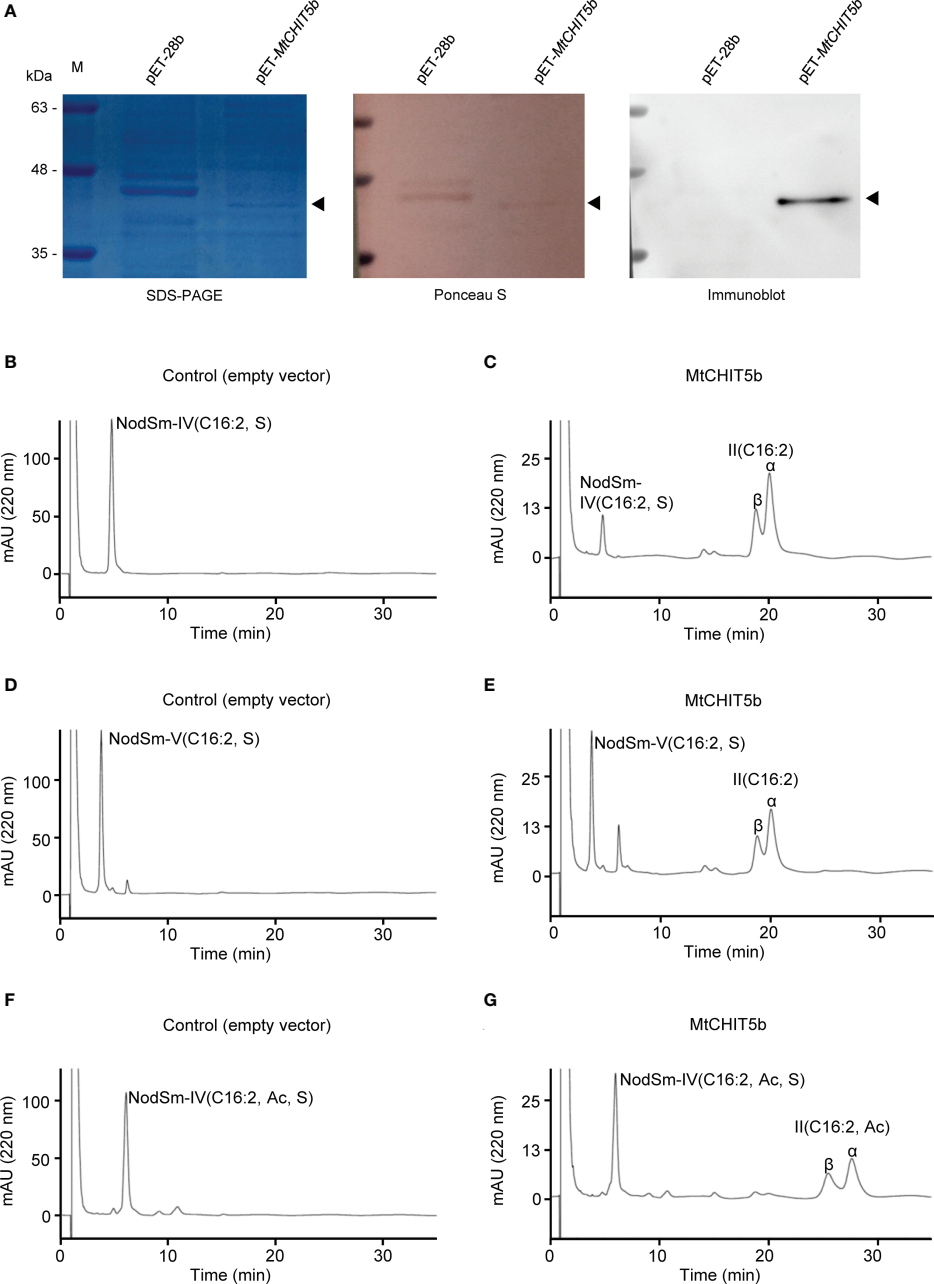
Preparation of recombinant MtCHIT5b protein and activity tests with Nod factor substrates. **(A)** Proteins eluted from nickel beads were separated on SDS-PAGE and stained with Coomassie Brilliant Blue R-250. Ponceau staining of the membrane served as a loading control. Immunoblot analysis was performed with an anti-His antibody **(B-G)** Activity tests with obtained MtCHIT5b (0.25 μg mL^-1^) and Nod factors (60 μM) in a 100-μL test system. After incubation (37 °C) for 12 h, the Nod factors and acylated cleavage products were extracted with *n*-butanol and analyzed by reverse-phase HPLC. **(B)** Incubation of control proteins from *E coli* carrying the empty vector pET-28b with NodSm-IV(C16:2, S). **(C)** Incubation of MtCHIT5b with NodSm-IV(C16:2, S) resulting in II(C16:2) formation. **(D)** Incubation of empty vector control proteins with NodSm-V(C16:2, S). **(E)** Incubation of MtCHIT5b with NodSm-V(C16:2, S) resulting in II(C16:2) formation. **(F)** Incubation of empty vector control proteins with NodSm-IV(C16:2, Ac, S). **(G)** Incubation of MtCHIT5b with NodSm-IV(C16:2, Ac, S) resulting in II(C16:2, Ac) formation.

The *M. truncatula* genes *MtCHIT5b* and *MtNFH1* show 75% amino acid sequence identity (without predicted signal peptide) (Zhang et al., 2016). *In vitro* activity tests with recombinant proteins expressed in *E. coli* showed that MtNFH1 can hydrolyze *S. meliloti* Nod factors into lipodisaccharides (Tian et al., 2013), while no detectable activity was found for MtCHIT5b in a previous study (Zhang et al., 2016). However, further enzyme tests in our laboratory indicated that His-tagged MtCHIT5b expressed in *E. coli* does not completely lack Nod factor cleaving activity. In these experiments, the previously constructed plasmid pET-*MtCHIT5b* and the empty vector pET-28b were freshly transformed into *E. coli* BL21 (DE3). The protein extracts were subjected to nickel affinity chromatography under native conditions using a lysis buffer of pH 7.5 to keep the pH significantly lower than the isoelectric point of His-tagged MtCHIT5b (pI 9.12). Proteins eluted from nickel beads with imidazole were supplemented with glycerol and adjusted to pH 7.0 with an acetate buffer to obtain a pH identical to the reaction buffer. SDS-PAGE gel analysis showed that the recombinant MtCHIT5b could be successfully eluted from the nickel column. The presence of the protein in the elution fraction was further confirmed by immunoblot analysis with an anti-His antibody (**Figure 1A**).

For Nod factor hydrolysis tests, recombinant MtCHIT5b (freshly prepared from *E. coli*) was incubated with tetrameric NodSm-IV(C16:2, S), pentameric NodSm-V(C16:2, S), or *O*-acetylated tetrameric NodSm-IV(C16:2, Ac, S). The Nod factor substrates and acylated cleavage products were then subjected to HPLC analysis using a C18 column. The obtained chromatograms showed that MtCHIT5b could release II(C16:2) from either NodSm-IV(C16:2, S) or NodSm-V(C16:2, S) (**Figures 1B-E**). Hydrolysis of NodSm-IV(C16:2, Ac, S) by MtCHIT5b resulted in formation of the II(C16:2, Ac) cleavage product (**Figures 1F, G**). The fractions containing II(C16:2) or II(C16:2, Ac) were collected and their molecular weights were confirmed by mass spectrometry (**Supplementary Figure 1**).

To obtain kinetic data, freshly prepared MtCHIT5b was incubated with a given Nod factor substrate at various concentrations. The protein amount of MtCHIT5b for these tests was determined by ImageJ software using a SDS-PAGE gel with different amounts of BSA as standards. Substrate-velocity curves are shown in **Supplementary Figure 2** and GraphPad Software was used to deduce corresponding Michaelis-Menten constants (*K*_m_ values). MtCHIT5b displayed a lower *K*_m_ value for NodSm-V(C16:2, S) than for NodSm-IV(C16:2, S) and NodSm-IV(C16:2, Ac, S), indicating a higher affinity of the enzyme to NodSm-V(C16:2, S). Furthermore, the activity of MtCHIT5b was calculated under substrate saturation conditions to obtain maximum velocity (V_max_) and catalytic rate constant (*k*_cat_) values. Highest *k*_cat_ and *k*_cat_/*K*_m_ values were obtained for NodSm-IV(C16:2, Ac, S), which is the most abundant *S. meliloti* Nod factor (**Table 1**). Collectively, these results show that MtCHIT5b is an enzyme that can hydrolyze NodSm-IV(C16:2, S), NodSm-V(C16:2, S) and NodSm-IV(C16:2, Ac, S) under the experimental conditions used in this study.

**Table 1:** Michaelis-Menten constants (*K*_m_), catalytic rate constants (*k*_cat_) and specific activity of MtCHIT5b for different Nod factor substrates.

Substrate	*K*_m_ (μM)	*k*_cat_ (s^-1^)	*k*_cat_ $K_{m}^{-1}$ (mM^-1^ s^-1^)	Enzyme activity (nkat mg^-1^)^b^
NodSm-IV(C16:2, S)	25.077 ± 3.994	0.457 ± 0.333	19.980 ± 17.470	11.113 ± 8.094
NodSm-V(C16:2, S)	3.578 ± 1.729^a^	0.157 ± 0.008	49.504 ± 17.761	3.825 ± 0.195
NodSm-IV(C16:2, Ac, S)	9.010 ± 2.872	0.786 ± 0.358	84.746 ± 18.725	19.098 ± 8.710

a Means ± SD of K_m_ and k_cat_ values (37 °C) deduced from kinetic data by using three independently purified enzyme preparations.

b Specific activity (activity per mg enzyme at V_max_).

### *MtCHIT5b* is expressed during the nodule symbiosis

**Figure 2:**
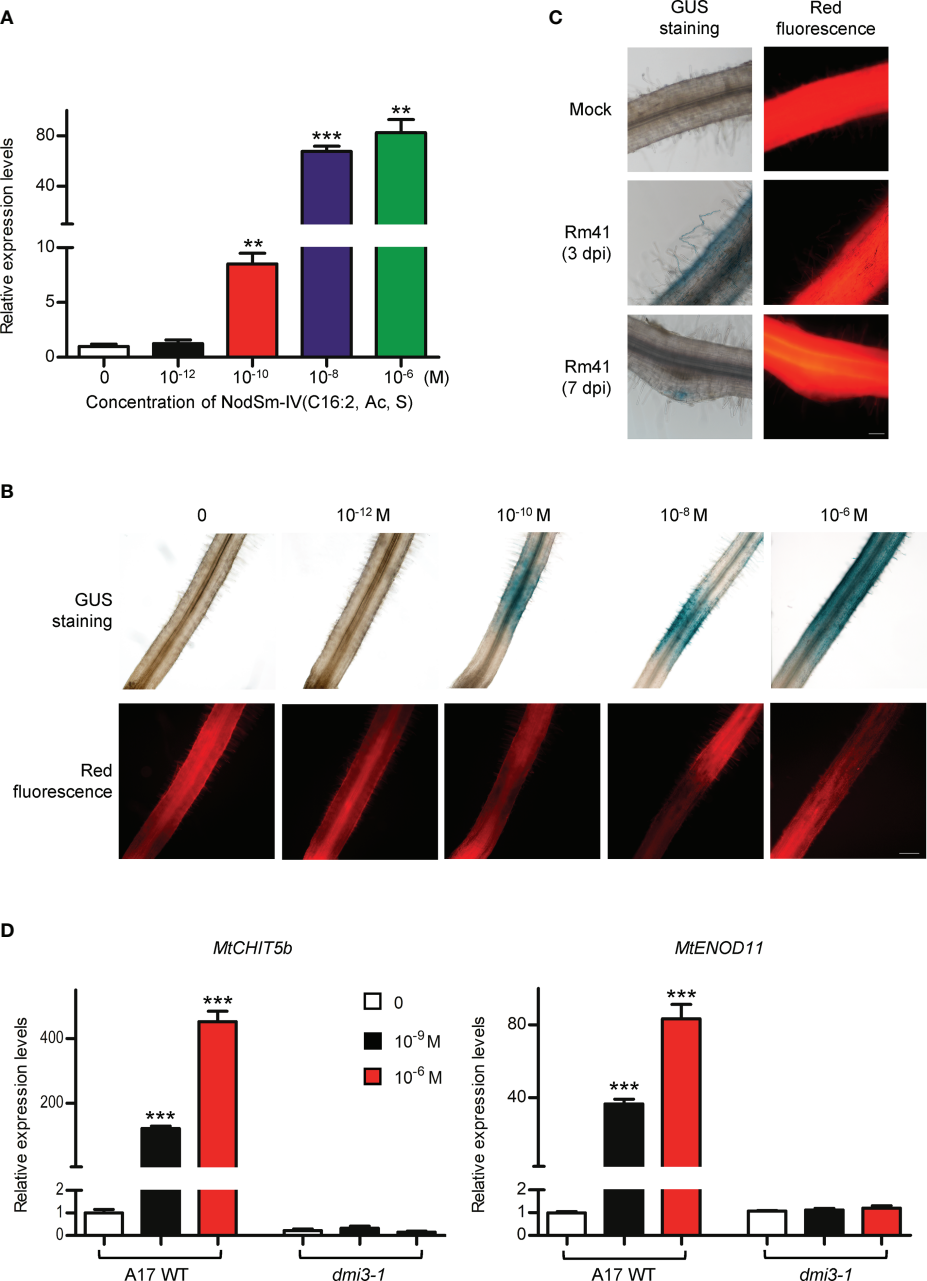
*MtCHIT5b* expression in roots of *M. truncatula* is induced by applied Nod factors and in response to *S. meliloti* inoculation. **(A)** R108 seedlings were treated with different concentrations of NodSm-IV(C16:2, Ac, S) dissolved in liquid BNM containing 0.5% (v/v) DMSO. After incubation (6 h in the dark), *MtCHIT5b* expression in roots was determined by RT-qPCR (7 seedlings per RNA extraction; *n* = 3). Data indicate means ± SE of normalized expression values (mean value of control set to one). The asterisks indicate significantly increased *MtCHIT5b* expression compared to control roots without Nod factor treatment (Student’s *t*-test; **, *P*< 0.01; ***, *P*< 0.001). **(B, C)** Promoter activity of *MtCHIT5b* in roots transformed with a *MtCHIT5b*p-*GUS* construct. Co-expressed RFP was used to identify transgenic roots. Plants were treated with different concentrations of NodSm-IV(C16:2, Ac, S) **(B)** or used for an inoculation experiment with *S. meliloti* Rm41 (mock-inoculation with 10 mM MgSO_4_; plants harvested at 3 dpi or 7 dpi) **(C)**. Transgenic roots showing red fluorescence were subjected to GUS staining. Bar = 200 μm in **(B)** and 100 μm in **(C)**. **(D)** Seedlings of Jemalong A17 wild-type plants and the nodulation signaling mutant *dmi3-1* were treated with indicated concentrations of NodSm-IV(C16:2, Ac, S) dissolved in liquid BNM containing 0.5% (v/v) DMSO. After incubation (6 h in the dark), *MtCHIT5b* and *MtENOD11* expression in roots was determined by RT-qPCR (7 seedlings per RNA extraction; *n* = 3). Data indicate means ± SE of normalized expression values (mean value of control set to one). The asterisks indicate significantly increased expression compared to control roots without Nod factor treatment (Student’s *t*-test; ***, *P*< 0.001).

*MtNFH1* gene expression in *M. truncatula* roots was found to be stimulated by root treatments with purified Nod factors (Cai et al., 2018). We therefore performed an RT-qPCR experiment to examine whether Nod factors show a similar effect on *MtCHIT5b* expression. In agreement with previously performed RNA sequencing data (Roux et al., 2014), *MtCHIT5b* expression was considerably up-regulated when roots were treated with different concentrations of NodSm-IV(C16:2, Ac, S) for 6 h (**Figure 2A**). A similar increase of transcripts was also measured for *MtNFH1* as reported earlier (Cai et al., 2018). In contrast, expression of *MtCHIT5a*, another class V chitinase gene of *M. truncatula*, was not affected by the Nod factor treatment (**Supplementary Figure 3**). Next, a 3 022-bp promoter sequence of *MtCHIT5b* was fused with the *GUS* gene to study tissue specific gene expression (*MtCHIT5b*p-*GUS* construct). In addition, the vector contained an *RFP* expression cassette for detection of the transgenic roots. Roots transformed with *A. rhizogenes* carrying the constructed binary vector were treated with Nod factors or inoculated with *S. meliloti* Rm41. GUS staining of untreated roots did not result in blue coloration reflecting *MtCHIT5b* promoter activity. However, strong blue coloration in young roots was observed when NodSm-IV(C16:2, Ac, S) was applied at a concentration range from 10^-6^ M to 10^-10^ M (**Figure 2B**). Similarly, inoculation with Rm41 caused induction of GUS activity in root hairs and the apex of nodule primordia (**Figure 2C**). Blue coloration was occasionally also observed in the central cylinder of roots treated with Nod factors or inoculated with Rm41. Nod factor treatments of *dmi3-1*, a non-nodulating *M. truncatula* Jemalong A17 mutant deficient in Nod factor signaling (Sagan et al., 1998), showed no effects on *MtCHIT5b* expression as determined by RT-qPCR analysis. Expression of the early nodulin gene *MtENOD11* was also analyzed in this experiment (**Figure 2D**). Overall, induction of *MtCHIT5b* expression correlated with rhizobial infection during early symbiotic stages and was dependent on Nod factor signaling.

**Figure 3:**
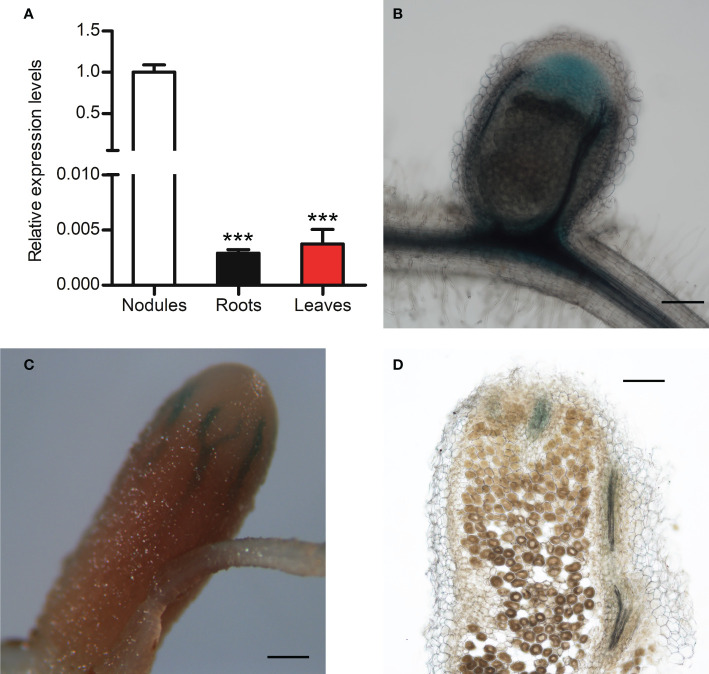
Analysis of *MtCHIT5b* expression in *M. truncatula* nodules. **(A)** RT-qPCR analysis of *MtCHIT5b* transcript levels in nodules of *M. truncatula* R108 inoculated with *S. meliloti* Rm41 and harvested at 3 wpi. For comparison, *MtCHIT5b* expression was also analyzed in roots and leaves of these plants. Data indicate means ± SE of normalized values (mean value of nodules set to one) from three independent RNA extractions (*n* = 3) (Student’s *t*-test; ***, P< 0.001). **(B-D)** GUS staining analysis of R108 nodules expressing the *MtCHIT5b*p-*GUS* construct at 3 wpi **(B)** and 4 wpi **(C, D)**. The picture in **(D)** shows a sectioned nodule. Bars = 200 μm in **(B, D)**; 500 μm in **(C)**.

To examine *MtCHIT5b* expression in nodules, plants were inoculated with Rm41 and nodules were harvested at 3 wpi. Isolated RNA was then used for RT-qPCR analysis. For comparison, *MtCHIT5b* expression in roots and leaves of these plants was also analyzed. Highest accumulation of *MtCHIT5b* transcripts was found for the nodules (**Figure 3A**). To further characterize *MtCHIT5b* expression in nodules, roots were transformed with the *MtCHIT5b*p-*GUS* construct and then inoculated with Rm41. GUS staining of nodules harvested at 3 wpi showed blue coloration in the apical region containing the infection zone. Nodules harvested at 4 wpi exhibited strong blue coloration in vascular bundles (**Figures 3B-D**). These results show that *MtCHIT5b* is expressed in mature nodules in accordance with previously reported RNA sequencing data from different nodule zones (Roux et al., 2014; **Supplementary Figure 4**).

### MtCHIT5b accumulates in the infection pocket of curled root hairs

**Figure 4:**
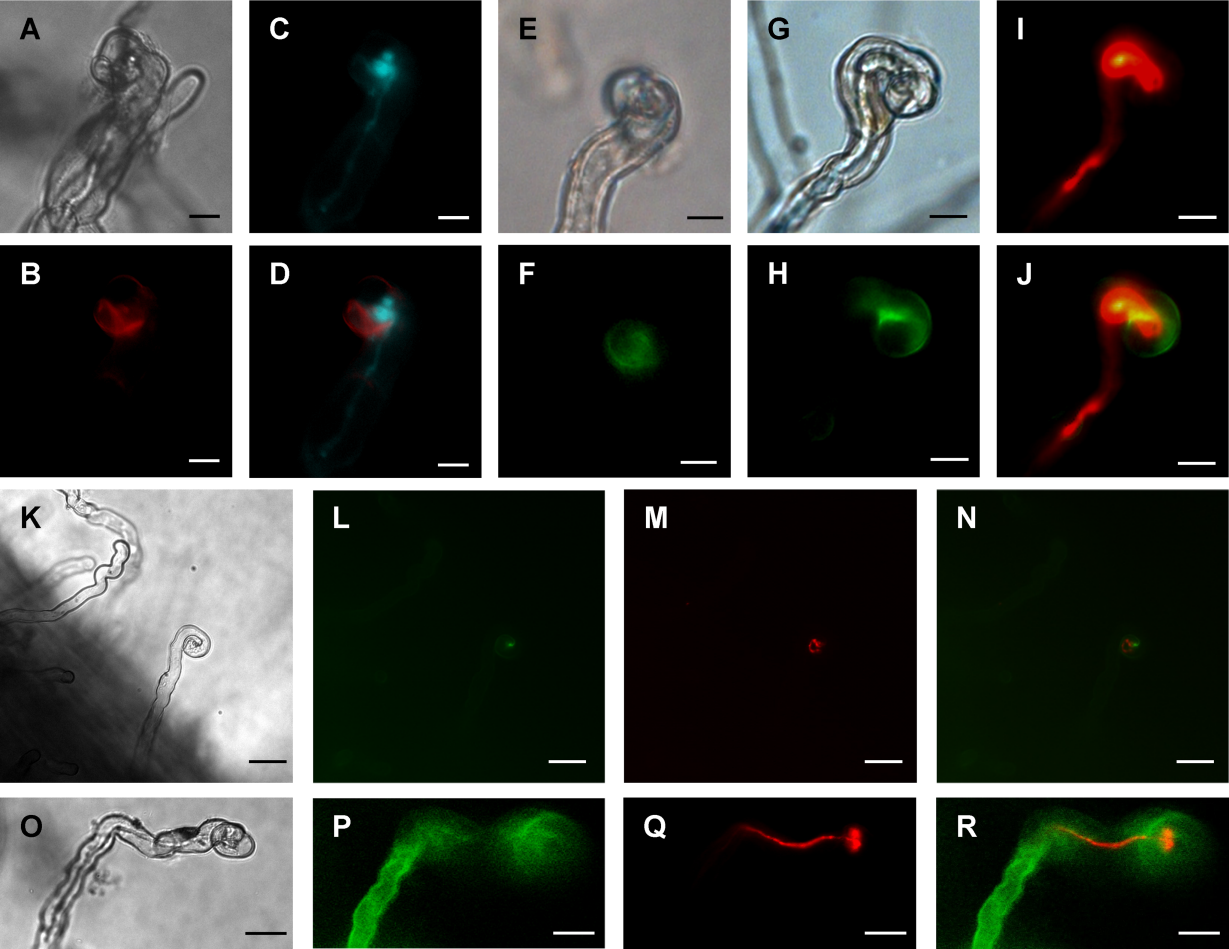
Subcellular localization of fluorescent MtCHIT5b in root hairs of *M. truncatula*. **(A-D)** Transgenic roots of R108 plants expressing *MtCHIT5b*:*mCherry* driven by a *Ubiquitin* gene promoter from *L. japonicus* were inoculated with *S. meliloti* Rm41-*mTFP1*. Root hairs were microscopically analyzed under bright field conditions **(A)**, for red fluorescence emission to detect the MtCHIT5b:mCherry fusion protein **(B)**, and for emission of teal fluorescent signals to visualize Rm41-*mTFP1* bacteria **(C)**. The overlay image **(D)** was obtained from **(B, C)** and indicates partial co-localization of MtCHIT5b:mCherry with teal fluorescent rhizobia in the infection pocket. **(E-N)** Transgenic roots expressing *MtCHIT5b*:*GFP* driven by the *MtCHIT5b* native promoter were inoculated with *S. meliloti* Rm41 **(E, F)** or *S. meliloti* Rm41-*mCherry* **(G-N)**. Root hairs were analyzed under bright field conditions **(E, G, K)**, for green fluorescent signals emitted by MtCHIT5b:GFP **(F, H, L)**, and for red fluorescent signals emitted by Rm41-*mCherry* **(I, M)**. The overlay images **(J, N)** indicate co-localization of MtCHIT5b:GFP with red fluorescent rhizobia in the infection pocket (J derived from H and I; N from L and M). **(O-R)** Similar analysis was performed for transgenic control roots expressing *GFP* driven by the CaMV 35S promoter. Roots were inoculated with *S. meliloti* Rm41-*mCherry* and root hairs were analyzed under bright field conditions **(O)** and for emission of green **(P)** or red **(Q)** fluorescent signals. The overlay image **(R)** was obtained from **(P, Q)**. Bars = 10 μm in **(A-J)** and 25 μm in **(K-R)**.

A gene construct encoding MtCHIT5b fused to the red fluorescent protein mCherry (driven by a *Ubiquitin* promoter from *L. japonicus*) was constitutively expressed in roots of *M. truncatula* to examine subcellular localization of MtCHIT5b. The transformed roots were then inoculated with *S. meliloti* Rm41 expressing the fluorescent protein mTFP1 (monomeric Teal Fluorescent Protein 1). Microscopy analysis of the inoculated roots showed that fluorescence signals emitted by the constitutively expressed MtCHIT5b:mCherry fusion protein are associated with curled root hairs, indicating that the protein was secreted into the rhizosphere and that the protein levels were increased in the infection pocket (**Figures 4A-D**). To substantiate these findings, roots were transformed with a construct encoding MtCHIT5b fused to green fluorescent protein (GFP), which was driven by a native promoter sequence of *MtCHIT5b* (3 033-bp sequence upstream of the ATG start codon). When roots were inoculated with Rm41, infected root hairs showed green fluorescence at their tips. Inoculation tests with Rm41 expressing mCherry indicated that fluorescence signals emitted by MtCHIT5b: GFP and mCherry partially co-localize in the region of the infection pocket, resulting in yellow signals in the overlay picture (**Figures 4E-J**). Neighboring root hairs without rhizobial infection did not show green fluorescence (**Figures 4K-N**). Control roots expressing *GFP* driven by the CaMV 35S promoter showed only faint background fluorescence in infection pockets when infected by rhizobia (**Figures 4O-R**). Overall, these findings indicate that MtCHIT5b is a secreted protein which comes into direct contact with Nod factor producing bacteria in the infection pocket. MtCHIT5b trafficking along the secretory pathway is also supported by bioinformatic prediction using SignalP-6.0 and the ProtComp 9.0 server (**Supplementary Figure 5**).

### MtCHIT5b contributes to Nod factor hydrolysis in the rhizosphere

To explore a possible MtCHIT5b function in symbiosis, R108 wild-type plants and a *MtCHIT5b* mutant were compared. The *Tnt1* retrotransposon mutant line NF16543 was obtained from the mutant collection at Oklahoma State University and homozygous mutant plants were identified by PCR analysis. The *Tnt1* insertion of these *chit5b* mutant plants is located 401-bp downstream of the ATG start codon of *MtCHIT5b*. In parallel, *nfh1-3b* mutant plants were PCR-selected from the NF11260 line, which was previously used to obtain *nfh1-3* (Cai et al., 2018). Compared to wild-type plants, *chit5b* plants showed significantly reduced *MtCHIT5b* transcript levels in roots treated with NodSm-IV(C16:2, S). Likewise, *MtNFH1* expression was reduced in *nfh1-3b* roots (**Supplementary Figure 6**).

**Figure 5:**
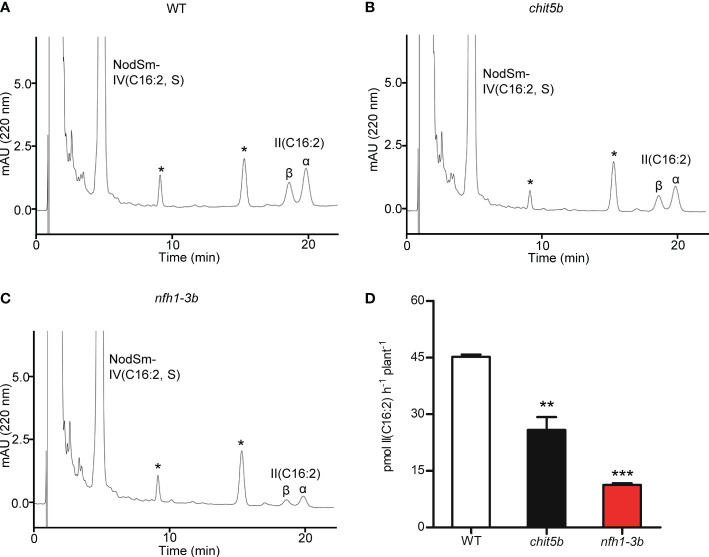
Nod factor degradation in the rhizosphere of *M. truncatula* wild-type, *chit5b* and *nfh1-3b* mutant plants. NodSm-IV (C16:2, S) hydrolysis was analyzed in the rhizosphere of R108 wild-type, *chit5b* and *nfh1-3b* seedlings. Roots were pretreated with 0.5 μM NodSm-IV (C16:2, S) for 18 h and then assayed for 2 h with 5.4 μM NodSm-IV (C16:2, S). Formation of II (C16:2) was analyzed by reverse-phase HPLC. **(A-C)** Representative chromatograms showing NodSm-IV(C16:2, S) and II (C16:2) from wild-type **(A)**, *chit5b* **(B)** and *nfh1-3b* **(C)** seedlings. Asterisks indicate nonidentified root compounds. **(D)** Hydrolytic activities deduced from HPLC chromatograms. Data indicate means ± SE (3 seedlings per sample; *n* = 3). The asterisks indicate significantly reduced cleavage activity in *chit5b* and *nfh1-3b* compared to wild-type plants (Student’s *t* test; **, *P*< 0.01; ***, *P*< 0.001).

The mutant plants were then characterized for their ability to cleave NodSm-IV(C16:2, S) in the rhizosphere. Roots of 5-day-old seedlings of wild-type, *chit5b* and *nfh1-3b* plants were pretreated with NodSm-IV(C16:2, S) for 18 h to induce hydrolase activity. The roots were then examined for rhizospheric Nod factor cleavage activity using the same Nod factor as substrate. After incubation, the seedlings were removed, and the incubation medium was extracted with *n*-butanol. The conversion of NodSm-IV(C16:2, S) into II(C16:2) was then detected by reverse-phase HPLC. Under the used test conditions, *chit5* and *nfh1-3b* plants showed both reduced Nod factor degradation activity as compared to wild-type plants. Quantitative analysis of HPLC peaks indicated that the sum of the *chit5* and *nfh1-3b* activities was comparable to the activity of wild-type plants. Thus, the secreted MtCHIT5b and MtNFH1 proteins appear to contribute additively to the hydrolysis of Nod factors in the rhizosphere (**Figure 5**).

The wild-type and mutant plants were then inoculated with *S. meliloti* Rm41 to determine their nodulation phenotype. Compared to wild-type plants, *chit5* and *nfh1-3b* showed a similar nodule number and nodule biomass at 3 wpi, indicating that these mutants were not impaired in nodule formation under the used test conditions (**Supplementary Figure 7**).

### Roots silenced in *MtCHIT5b*/*MtNFH1* show reduced nodulation

**Figure 6:**
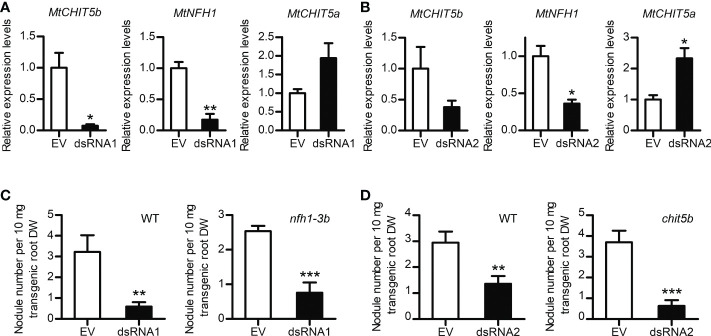
Nodulation phenotype of *M. truncatula* roots silenced in *MtCHIT5b*/*MtNFH1* expression by the dsRNA1 and dsRNA2 constructs. **(A, B)** RT-qPCR analysis of *MtCHIT5b, MtNFH1* and *MtCHIT5a* expression in R108 wild-type roots transformed with the dsRNA1 construct **(A)**. or the dsRNA2 construct **(B)**. Roots transformed with the empty vector (EV) containing *RFP* alone served as a control. Plants were harvested at 17 days post *A. rhizogenes*-mediated transformation and co-expression of *RFP* allowed identification of transgenic roots. Data indicate means ± SE of normalized values (*n* = 3; mean expression value of the empty vector control set to one) (Student’s *t*-test; *, *P*< 0.05; **, *P<* 0.01). **(C, D)** Nodule formation on roots of R108 wild-type (WT), *nfh1-3b* or *chit5b* mutant plants transformed with the dsRNA1 construct **(C)** or the dsRNA2 construct **(D)**. Roots transformed with the EV served as a control. Plants with red fluorescent roots were inoculated with *S. meliloti* Rm41. At the time of harvest (4 wpi), the nodule frequency per root dry weight (DW) was determined for each plant. Data indicate means ± SE (Student’s *t* test; **, *P*< 0.01; ***, *P*< 0.001). Data shown in panel **(C)** were obtained from 16 transformed WT roots (EV, *n* = 7; dsRNA1, *n* = 9) and from 20 transformed *nfh1-3b* mutant roots (EV, *n* = 9; dsRNA1, *n* = 11). Data shown in panel D were obtained from 17 transformed WT roots (EV, *n* = 9; dsRNA2, *n* = 8) and 22 transformed *chit5b* mutant roots (EV, *n* = 10; dsRNA2, *n* = 12).

An RNA interference (RNAi) approach was used to silence *MtCHIT5b* and *MtNFH1* in roots of *M. truncatula* plants. The coding sequences of *MtCHIT5b* and *MtNFH1* show 80% nucleotide identity at the mRNA level. RNAi-silenced roots were generated by *A. rhizogenes*-mediated transformation using a binary vector containing an RNAi construct derived from the *MtCHIT5b* sequence in the sense and antisense orientations (named dsRNA1). In an additional experiment, an RNAi construct derived from the *MtNFH1* sequence (named dsRNA2) was used (Tian et al., 2013). For detection of transformed roots, the binary vectors also contained an *RFP* expression cassette. RT-qPCR analysis of red fluorescent roots showed that expression of *MtCHIT5b* and *MtNFH1* in wild-type plants was reduced in roots transformed with the dsRNA1 or dsRNA2 constructs. Due to a possible compensation effect, a slightly higher expression level in these roots was determined for the chitinase gene *MtCHIT5a* (Zhang et al., 2016), which is most related to *MtCHIT5b/MtNFH1* (**Figures 6A, B**). Moreover, when treated with Nod factors, dsRNA2-transformed roots showed reduced transcript levels not only for *MtCHIT5b* but also for *MtENOD11*, suggesting that Nod factor signaling was partially impaired (**Supplementary Figure 8**). Wild-type and *nfh1-3b* roots, both transformed with the dsRNA1 construct, were then used for nodulation tests with *S. meliloti* Rm41. Non-transgenic roots lacking co-expressed *RFP* were identified by fluorescence microscopy and removed prior to inoculation. Compared to the empty vector control, the nodule frequency (number of nodules per root biomass) was 5.4-fold lower for dsRNA1-transformed wild-type roots and 3.4-fold lower for dsRNA1-transformed *nfh1-3b* roots (**Figure 6C**). Similarly, a 2.2-fold lower nodule frequency was observed for dsRNA2-transformed wild-type roots and a 5.9-fold lower one for dsRNA2-transformed *chit5b* roots (**Figure 6D**). When calculated on a whole-plant basis, nodule numbers and nodule biomass values were significantly reduced in dsRNA1- or dsRNA2-transformed roots of all examined genotypes (**Supplementary Figure 9**). Overall, the performed RNAi experiments indicate that simultaneous silencing of *MtCHIT5b* and *MtNFH1* impairs nodule formation in *M. truncatula* plants.

### *MtCHIT5b*-overexpressing roots also show reduced nodule formation

**Figure 7:**
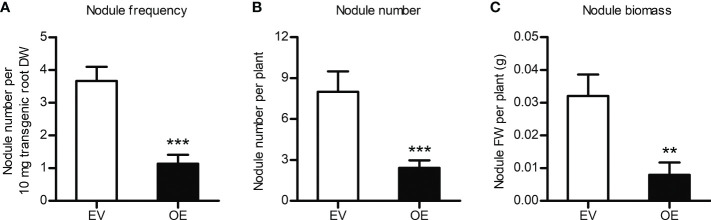
Constitutive overexpression of *MtCHIT5b* impairs nodulation of *M. truncatula*. R108 plants constitutively overexpressing *MtCHIT5b* in roots (OE) were obtained by *A rhizogenes*-mediated transformation. Plants with red fluorescent roots (co-expression of *RFP*) were inoculated with *S. meliloti* Rm41 and harvested at 4 wpi. Plants transformed with the empty vector containing *RFP* alone (EV) served as a control. Data indicate means ± SE (EV, *n* = 8; OE, *n* = 12) (Student’s *t* test; **, *P*< 0.01; ***, *P*< 0.001). **(A)** Number of formed nodules per root dry weight (DW). **(B)** Number of formed nodules per plant. **(C)** Biomass (fresh weight, FW) of formed nodules per plant.

To study the effects of *MtCHIT5b*-overexpression, a binary vector containing the *MtCHIT5b* coding sequence driven by a *Ubiquitin* promoter from *L. japonicus* was constructed. *A. rhizogenes* strains carrying this vector or the empty vector (containing *RFP*) were then used to transform roots of *M. truncatula* R108 plants. RT-qPCR analysis indicated constitutive overexpression of *MtCHIT5b* in red fluorescent roots while expression of *MtNFH1* and *MtCHIT5a* was not altered (**Supplementary Figure 10A-C**). When treated with Nod factors, the transformed roots also exhibited strong overexpression of *MtCHIT5b*, whereas transcript levels of *MtENOD11* were lower than in control plants (**Supplementary Figure 10D-E**). These findings suggest that the applied Nod factors were rapidly degraded by the *MtCHIT5b*-overexpressing roots. For nodulation tests with *S. meliloti* Rm41, non-transgenic roots (lacking co-expressed RFP) were removed before inoculation. Compared to the empty vector control, the nodule frequency (number of nodules per root biomass) was reduced in plants with *MtCHIT5b*-overexpressing roots. When calculated on a whole-plant basis, plants with roots overexpressing *MtCHIT5b* formed fewer nodules and the nodule biomass was also lower (**Figure 7**). Thus, overexpression of *MtCHIT5b* and *MtCHIT5b*/*MtNFH1* silencing in *M. truncatula* roots resulted in a similar symbiotic phenotype.

## Discussion

We report in this study that MtCHIT5b expressed in *E. coli* possesses Nod factor-cleaving activity under newly established test conditions. Like recombinant MtNFH1 (Tian et al., 2013), MtCHIT5b was found to release lipodisaccharides from *S. meliloti* Nod factors *in vitro*. Detection of such an activity was surprising because MtCHIT5b expressed in *E. coli* was reported to be only active when the serine at the predicted substrate binding cleft is substituted to proline (Zhang et al., 2016). In the present study, the His-tagged MtCHIT5b protein (pI 9.12) was extracted from *E. coli* cells at pH 7.5, while pH 8.0 was used previously (Zhang et al., 2016). Moreover, His-tagged MtCHIT5b (eluted from the nickel column with imidazole) was supplemented with glycerol and immediately adjusted to pH 7.0 with acetate buffer. Subsequent Nod factor hydrolysis was performed in a potassium phosphate buffer (pH 7.0), whereas a sodium acetate buffer (pH 5.0) was used previously (Zhang et al., 2016). In comparison with MtNFH1 (Tian et al., 2013), MtCHIT5b showed lower Nod factor hydrolase activity but displayed higher affinity to Nod factors (lower *K*_m_ values) under the test conditions used in this work.

Our tests with Nod factors added to roots of *M. truncatula* seedlings indicated that MtCHIT5b contributes to Nod factor hydrolysis in the rhizosphere, particularly when roots of 5-day-old seedlings are incubated in 1-mL syringes filled with BNM. Previous studies were performed with younger seedlings and used Jensen medium for such tests (Staehelin et al., 1994b; Staehelin et al., 1995; Tian et al., 2013; Cai et al., 2018). The sum of rhizospheric Nod factor hydrolase activity of the *chit5b* and *nfh1-3b* mutants could reach the activity level of wild-type plants, suggesting that MtCHIT5b and MtNFH1 are the two major Nod factor-cleaving enzymes in the rhizosphere. In this context, it is worth noting that no extracellular Nod factor cleaving activity was observed for roots of young *nfh1-3* mutant seedlings treated with Nod factors (Cai et al., 2018), suggesting that *MtCHIT5b* expression is weak at such an early stage.

MtCHIT5b and MtNFH1 appear to have a similar gene expression pattern and subcellular localization. A Nod factor treatment of *M. truncatula* roots resulted in increased *MtCHIT5b* transcript accumulation within few hours, indicating that expression of *MtCHIT5b* is regulated by Nod factor signaling as reported for *MtNFH1* previously (Cai et al., 2018). Induction of *MtCHIT5b* expression by Nod factors was stronger than that of *MtNFH1* in our experiment (**Figure 2A**; **Supplementary Figure 3**), suggesting that the promoters of these genes differ in Nod factor-responsive elements. Furthermore, the results of our promoter-*GUS* fusion analysis indicated that seedlings inoculated with *S. meliloti* exhibit strongly induced *MtCHIT5b* promoter activity in emerging root hairs and on the tips of nodule primordia. Microscopic analysis of *S. meliloti-*infected seedlings expressing MtCHIT5b fused to a fluorescent protein showed strong fluorescence signals in the infection pocket of a curled root hair. A similar subcellular localization was observed for MtNFH1 (Cai et al., 2018). Thus, both enzymes appear to be co-localized with the rhizobial microcolony in the infection pocket. These observations suggest that Nod factor levels in the infection pocket are simultaneously reduced by both enzymes. In older *M. truncatula* plants, formed nodules showed relatively high expression levels of *MtCHIT5b*. These findings are largely in line with the RNA sequencing results obtained by Roux et al. (2014). The accumulation of *MtCHIT5b* transcripts in nodules indicates that the enzyme may participate in symbiotic processes in the infection zone of mature nodules.

RNAi-mediated silencing of *MtCHIT5b*/*MtNFH1* led to a considerable reduction in nodule formation (**Figure 6**). Because of the high similarity between the *MtCHIT5b*/*MtNFH1* nucleotide sequences, the dsRNA1 and dsRNA2 constructs caused silencing of both genes in the transformed roots. On the other hand, nodulation tests with *chit5*, *nfh1-3b* and wild-type plants showed no obvious differences, indicating that knockout of a single hydrolase gene was not sufficient to affect nodule formation under the test conditions used (**Supplementary Figure 7**). Overall, we conclude that MtCHIT5b and MtNFH1 show additive effects on Nod factor hydrolysis in the rhizosphere and cooperatively promote nodule formation. In other words, MtCHIT5b and MtNFH1 act redundantly with respect to nodule formation. We hypothesize that high concentrations of Nod factors impair nodule formation and that inactivation of excess amounts of Nod factors by MtCHIT5b and MtNFH1 promotes nodule formation. A permanent activation of Nod factor signaling by too high Nod factor concentrations could possibly impair establishment of symbiosis. Furthermore, it cannot be ruled out that high concentrations of Nod factors induce plant immunity and autoregulation responses which are known to negatively affect nodulation (Cao et al., 2017; Cai et al., 2018; Li et al., 2022). The experiments shown in **Figure 2** indicate that *MtCHIT5b* expression was stimulated by inoculation with rhizobia and by Nod factor concentrations as low as 10^-10^ M. These findings indicate that the concentration of Nod factors produced by rhizobia in the rhizosphere was at least 10^-10^ M. We suggest that Nod factor levels in the infection pocket are much higher.

While *MtCHIT5b* and *MtNFH1* must be co-silenced to negatively affect nodule formation in *M. truncatula*, mutation of the single chitinase gene *LjCHIT5* was sufficient to impair nodule formation in *L. japonicus* (Malolepszy et al., 2018). LjCHIT5 can apparently hydrolyze Nod factors of *M. loti* (Malolepszy et al., 2018) whereas cleavage of *S. meliloti* Nod factors by recombinant LjCHIT5 was not observed *in vitro* (Zhang et al., 2016). These data indicate that Nod factor cleaving enzymes in different legumes possess different substrate specificities. It is worth noting in this context that *LjCHIT5* is more related to *MtCHIT5a* than to the *MtCHIT5b*/*MtNFH1* genes, which belong to a separate clade in a constructed phylogenetic tree (**Supplementary Figure 11**). Thus, it appears that *M. truncatula* and *L. japonicus* have recruited different class V chitinase genes for nodule symbiosis during evolution.

Roots constitutively overexpressing *MtCHIT5b* showed reduced nodule formation in our study. Overexpression of *MtCHIT5b* likely caused excessive hydrolysis of Nod factors, resulting in too low signal concentrations and thus reduced bacterial infection. Inoculation of plants with rhizobia expressing a bacterial chitinase gene and thus producing less Nod factor is another strategy to examine the symbiotic role of Nod factor levels. Using this approach, alfalfa plants inoculated with *S. meliloti* expressing the *chiB* gene of *Serratia marcescens* showed delayed nodulation (Krishnan et al., 1999). Taken together, these results indicate that the amount of Nod factors required for infection and subsequent nodulation must reach a minimum level.

In conclusion, the data obtained in this work show that MtCHIT5b contributes to hydrolysis of *S. meliloti* Nod factors. MtCHIT5b and MtNFH1 cooperate to adjust Nod factors to an optimal concentration range for nodulation.

## Data availability statement

The original contributions presented in the study are included in the article/**Supplementary Material**. Further inquiries can be directed to the corresponding authors.

## Author contributions

R-JL, C-XZ, S-YF and Y-HW performed research. JW and KM screened for *Tnt1* insertion mutants and provided seeds. R-JL, C-XZ, S-YF, Y-HW, Z-PX and CS analyzed data. R-JL, Z-PX and CS conceived the work and wrote the paper. All authors contributed to the article and approved the submitted version.

## Funding

This work was financially supported by the National Natural Science Foundation of China (grants 32161133026 and 31670241), the Science Foundation of the State Key Laboratory of Biocontrol and the Guangdong Provincial Key Laboratory of Plant Resources (grant 2020B1212060027).

## Acknowledgments

We are grateful to Yu-Feng Wu, Qi Sun, Wen-Hui Hu, Chun-Lian Li, Christian Wagner and Yan Wang (Sun Yat-sen University) for their help with various aspects of this work. We thank Eva Kondorosi (Biological Research Center, Hungarian Academy of Sciences, Szeged, Hungary) for *S. meliloti* strain 1021 (pEK327) and Clare Gough (INRAE-CNRS, Castanet-Tolosan, France) for the *dmi3-1* mutant.

## Conflict of interest

The authors declare that the research was conducted in the absence of any commercial or financial relationships that could be construed as a potential conflict of interest.

## Publisher’s note

All claims expressed in this article are solely those of the authors and do not necessarily represent those of their affiliated organizations, or those of the publisher, the editors and the reviewers. Any product that may be evaluated in this article, or claim that may be made by its manufacturer, is not guaranteed or endorsed by the publisher.
