# Supplementary material for: The Medicago truncatula hydrolase MtCHIT5b degrades Nod factors of Sinorhizobium meliloti and cooperates with MtNFH1 to regulate the nodule symbiosis
Source: Front Plant Sci. 2022 Nov 17;13:1034230. doi: 10.3389/fpls.2022.1034230 (PMC9712974; doi:10.3389/fpls.2022.1034230)
Supplement: Supplementary file 1 [file DataSheet_1.pdf]

Supplementary Figures

The *Medicago truncatula* hydrolase MtCHIT5b degrades Nod factors of *Sinorhizobium meliloti* and cooperates with MtNFH1 to regulate the nodule symbiosis

Ru-Jie Li, Chun-Xiao Zhang, Sheng-Yao Fan, Yi-Han Wang, Jiangqi Wen, Kirankumar S. Mysore, Zhi-Ping Xie and Christian Staehelin

This file includes Supplementary Figures 1 to 11:

SUPPLEMENTARY FIGURE 1 Chemical structures and mass spectrometry analysis of II(C16:2) and II(C16:2, Ac).
SUPPLEMENTARY FIGURE 2 Velocity versus Nod factor substrate concentration curves of MtCHIT5b.
SUPPLEMENTARY FIGURE 3 RT-qPCR expression analysis for *MtNFH1* and *MtCHIT5a* in *M. truncatula* roots treated with different concentrations of NodSm-IV(C16:2, Ac, S).
SUPPLEMENTARY FIGURE 4 *MtCHIT5b* expression data for different nodule zones downloaded from the Symbimics homepage.
SUPPLEMENTARY FIGURE 5 Signal peptide and subcellular localization prediction for MtCHIT5b.
SUPPLEMENTARY FIGURE 6 Characterization of the *Tnt1* insertion mutants *chit5b* and *nfh1-3b*.
SUPPLEMENTARY FIGURE 7 Nodule formation of *chit5b* and *nfh1-3b* mutant plants inoculated with *S. meliloti* Rm41.
SUPPLEMENTARY FIGURE 8 Expression analysis of *MtCHIT5b* and *MtENOD11* in dsRNA2-transformed *M. truncatula* roots treated with NodSm-IV(C16:2, S).
SUPPLEMENTARY FIGURE 9 Nodule parameters on a whole-plant basis obtained from nodulated *M. truncatula* roots transformed with dsRNA1 or dsRNA2 constructs.
SUPPLEMENTARY FIGURE 10 RT-qPCR analysis of *M. truncatula* roots constitutively overexpressing *MtCHIT5b*.
SUPPLEMENTARY FIGURE 11 Phylogenetic analysis of leguminous class V chitinase protein sequences.

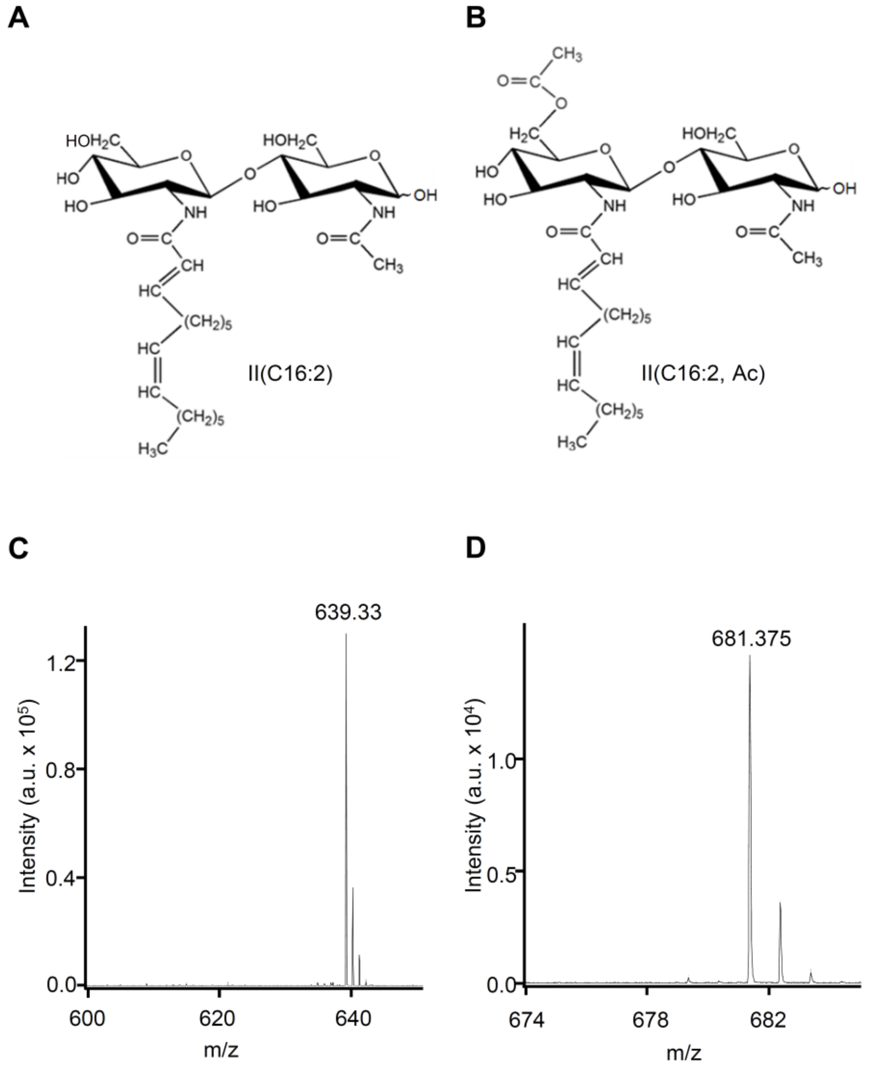

SUPPLEMENTARY FIGURE 1 Chemical structures and mass spectrometry analysis of II(C16:2) and II(C16:2, Ac).
(A) Chemical structure of the lipodisaccharide II(C16:2) consisting of two sugar residues with a C16:2 fatty acyl chain at the non-reducing end. **(B)** Chemical structure of the acetylated lipodisaccharide II(C16:2, Ac). **(C)** Positive-ion mode MALDI-TOF mass spectrometry analysis of purified II(C16:2). The major peak with m/z 639.33 corresponds to II(C16:2) with a sodium adduct (predicted m/z 639.83). **(D)** Positive-ion mode MALDI-TOF mass spectrometry analysis of purified II(C16:2, Ac). The major peak with m/z 681.375 corresponds to II(C16:2, Ac) with a sodium adduct (predicted m/z 681.87).

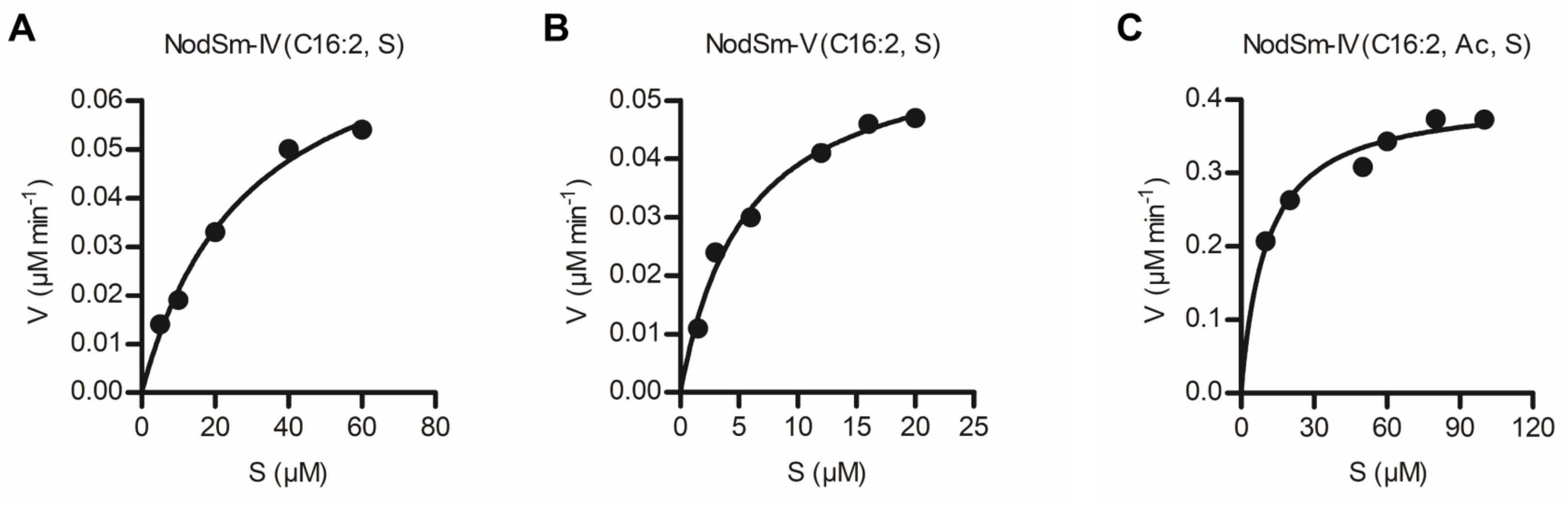

SUPPLEMENTARY FIGURE 2 Velocity versus Nod factor substrate concentration curves of MtCHIT5b.

Recombinant MtCHIT5b protein (0.25 μg mL^{-1}) was incubated at 37 ºC with Nod factors at different concentrations. Lipodisaccharide formation was quantified from areas of II(C16:2) and II(C16:2, Ac) peaks of HPLC chromatograms. **(A)** NodSm-IV(C16:2, S) as substrate. **(B)** NodSm-V(C16:2, S) as substrate. **(C)** NodSm-IV(C16:2, Ac, S) as substrate.

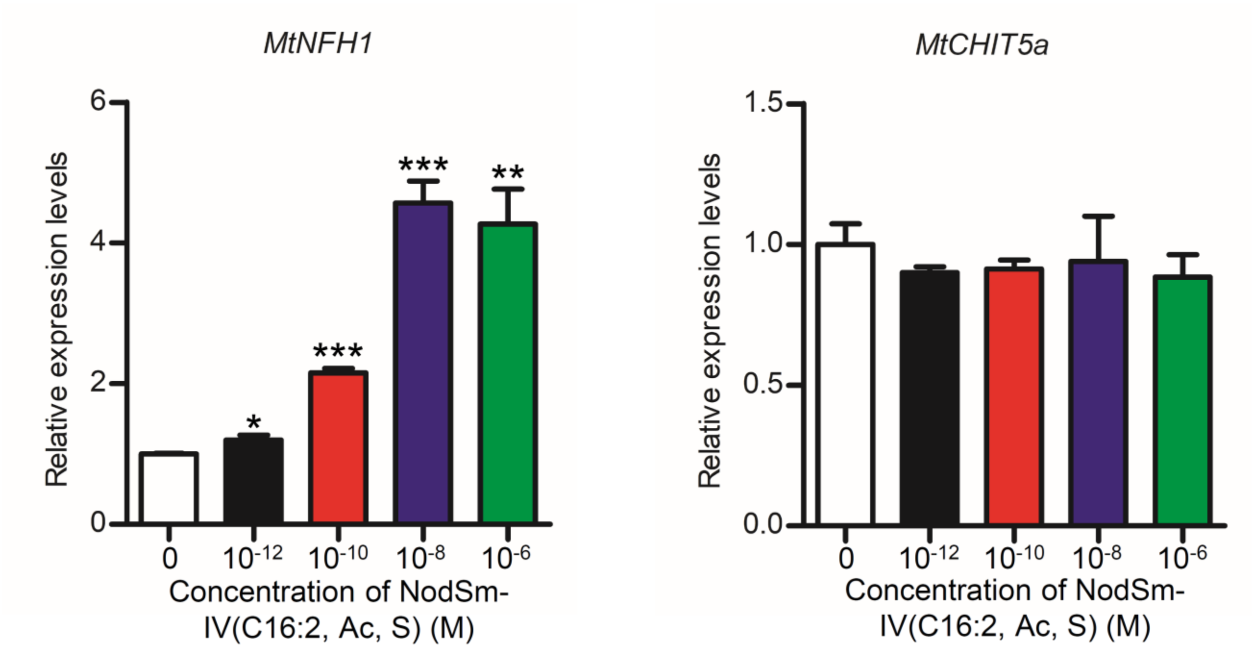

SUPPLEMENTARY FIGURE 3 RT-qPCR expression analysis for *MtNFH1* and *MtCHIT5a* in *M. truncatula* roots treated with different concentrations of NodSm-IV(C16:2, Ac, S).

RT-qPCR was performed to quantify *MtNFH1* and *MtCHIT5a* transcript levels in roots of R108 (wild-type) plants treated with liquid BNM containing 0.5% (v/v) DMSO alone or supplemented with different concentrations of NodSm-IV(C16:2, Ac, S). After incubation for 6 h, roots were harvested and 7 seedlings were used for one RNA extraction (n = 3). Data indicate means ± SE of normalized expression values (mean value of control set to one). A statistically significant increase of *MtNFH1* transcripts in Nod factor treated roots compared to control roots is marked by asterisks (Student's t-test; *, $P < 0.05$; **, $P < 0.01$; ***, $P < 0.001$).

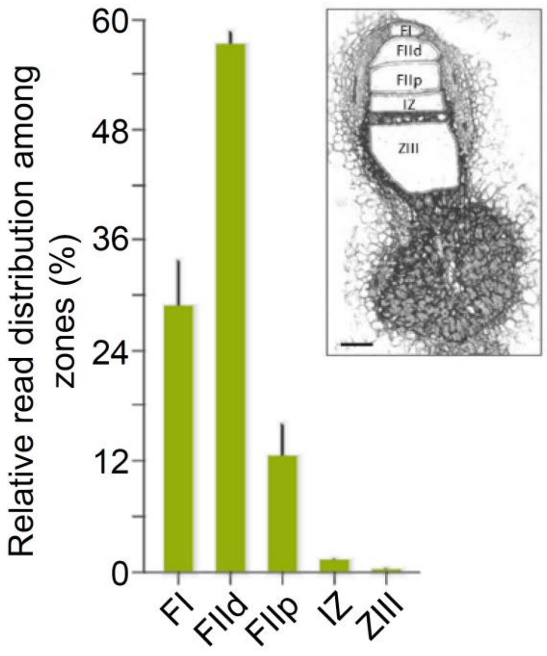

SUPPLEMENTARY FIGURE 4 *MtCHIT5b* expression data for different nodule zones downloaded from the Symbimics homepage.
RNA samples from *M. truncatula* (ecotype A17) nodules induced by *S. meliloti* GMI11495 (15 dpi) were obtained by a laser-capture microdissection method and used for RNA sequencing (Roux et al., 2014). The photograph (inset) illustrates the different nodule zones (from Fig. 1A in Roux et al., 2014; FI, fraction I; FIId, distal fraction; FIIp, proximal fraction II; IZ, interzone; ZIII, zone III; bar = 100 µm). The *MtCHIT5b* expression data (Medtr4g117000) were downloaded from the Symbimics homepage. The layout of the graph was modified.
References: Roux, B., Rodde, N., Jardinaud, M.F., Timmers, T., Sauviac, L., Cottret, L., Carrerè, S., Sallet, E., Courcelle, E., Moreau, S., Debellé, F., Capela, D., de Carvalho-Niebel, F., Gouzy, J., Bruand, C., and Gamas, P. (2014). An integrated analysis of plant and bacterial gene expression in symbiotic root nodules using laser-capture microdissection coupled to RNA sequencing. *Plant J.* 77**:** 817–837. Symbimics homepage (INRA/CNRS, Castanet-Tolosan, France): https://iant.toulouse.inra.fr/symbimics.

A

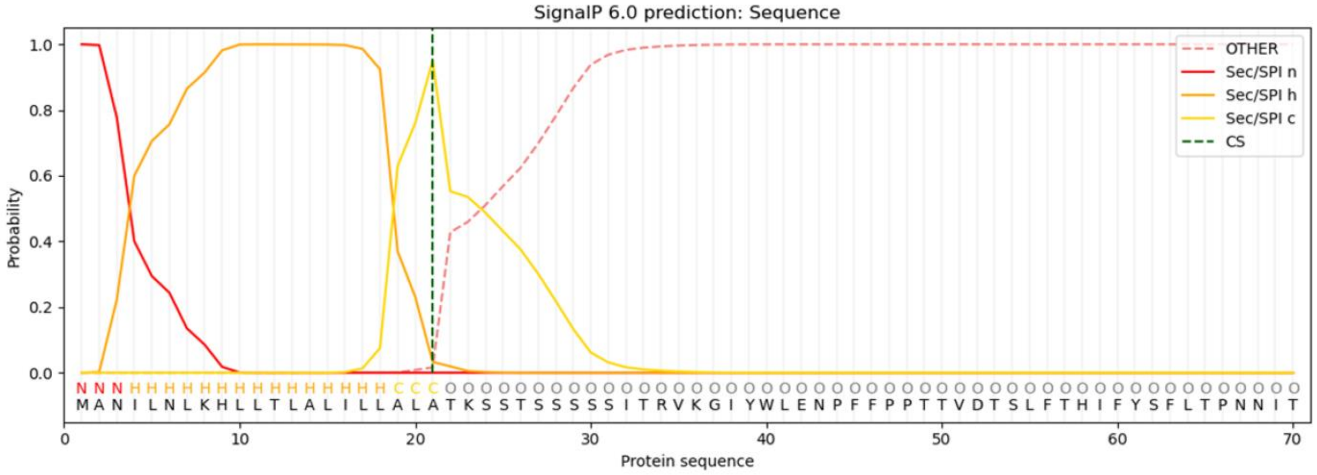

B

```
5724 multiple located sequences are accepted
ProtComp Version 9.0. Identifying sub-cellular location (Plant)
Seq name:  test sequence, Length=379
Significant similarity by DBSCAN-P - NONE
Predicted by Neural Nets - Extracellular (Secreted) with score    0.9
Integral Prediction of protein location: Extracellular (Secreted) with score    2.3
Location weights:     LocDB / PotLocDB / Neural Nets / Pentamers / Integral
 Nuclear                0.0 /      0.0 /        0.00 /      0.00 /     0.00
 Plasma membrane        0.0 /      0.0 /        0.89 /      0.44 /     0.00
 Extracellular          0.0 /      0.0 /        0.89 /      1.28 /     2.35
 Cytoplasmic            0.0 /      0.0 /        0.00 /      0.47 /     1.33
 Mitochondrial          0.0 /      0.0 /        0.00 /      2.35 /     1.69
 Endoplasm. retic.      0.0 /      0.0 /        0.00 /      1.07 /     0.55
 Peroxisomal            0.0 /      0.0 /        0.89 /      0.00 /     2.09
 Golgi                  0.0 /      0.0 /        0.34 /      0.07 /     1.47
 Chloroplast            0.0 /      0.0 /        0.00 /      0.24 /     0.54
 Vacuolar               0.0 /      0.0 /        0.00 /      0.00 /     0.00
```

SUPPLEMENTARY FIGURE 5 Signal peptide and subcellular localization prediction for MtCHIT5b.
(A) Results of signal peptide prediction performed with SignalP-6.0. The cleavage site is between 21 and 22, namely ALA-TK. The probability is 0.950756. **(B)** Results of subcellular localization prediction performed with ProtComp 9.0. MtCHIT5b is predicted to be secreted.

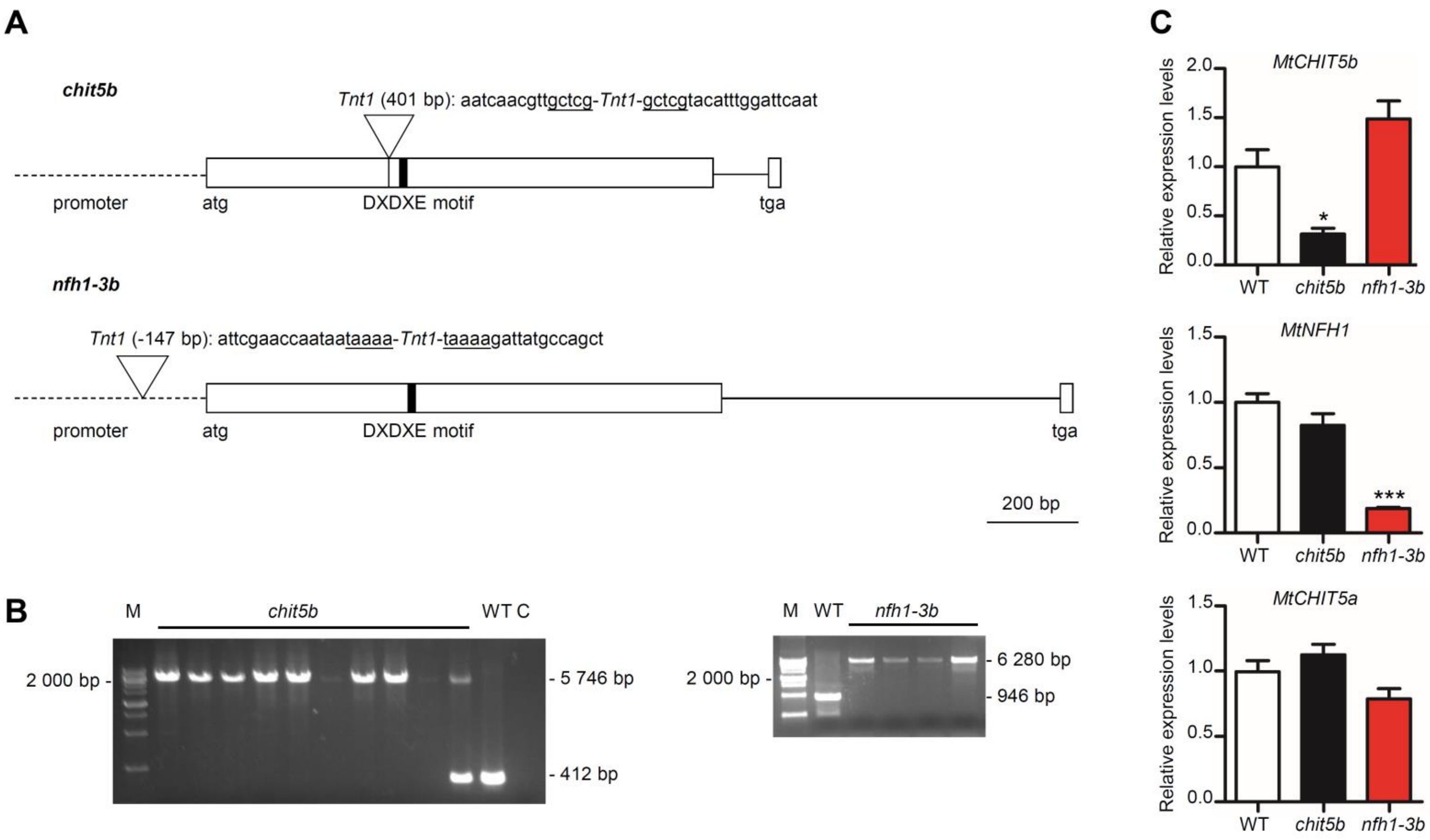

SUPPLEMENTARY FIGURE 6 Characterization of the *Tnt1* insertion mutants *chit5b* and *nfh1-3b*.
(A) *Tnt1* insertion sites of the *M. truncatula* R108 mutants *chit5b* (NF16543) and *nfh1-3b* (NF11260). The rectangles correspond to the coding region and the horizontal lines indicate an intron. **(B)** PCR analysis to obtain homozygous *chit5b* and *nfh1-3b* mutant plants. Primers designed from the flanking regions of the *Tnt1* insertion site were used to amplify the shown amplicons using genomic DNA from *chit5b* candidates (derived from NF16543) or *nfh1-3b* candidates (derived from NF11260) as a template. Genomic DNA of R108 wild-type (WT) plants was used as a template to amplify the 412-bp band (left) and the 946-bp band (right). The upper bands correspond to amplified *Tnt1* with flanking genomic DNA. Amplification of a unique upper band of expected size (5 746-bp for *chit5b*; 6 280-bp for *nfh1-3b*) allowed the identification of homozygous mutant plants. **(C)** RT-qPCR analysis of *MtCHIT5b*, *MtNFH1* and *MtCHIT5a* transcript levels in roots of R108 wild-type, *chit5b* and *nfh1-3b* plants. Seedlings were treated with 10^{-7} M NodSm-IV(C16:2, S) for 6 h. Roots from 7 seedlings were used for each RNA sample (n = 3). Data indicate means ± SE of normalized expression values (mean value of wild-type set to one) (Student's t-test; *, $P < 0.05$; ***, $P < 0.001$).

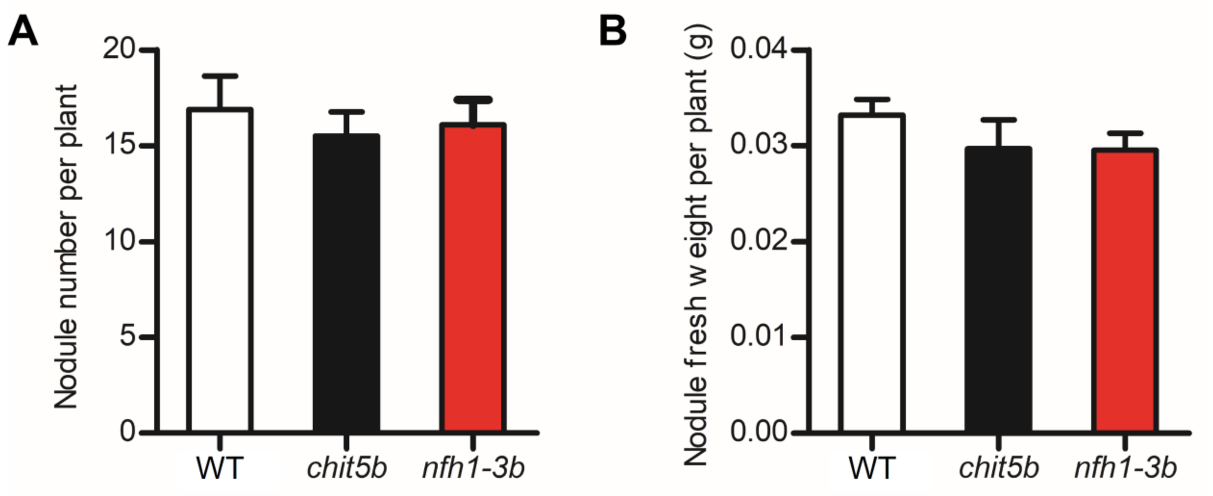

SUPPLEMENTARY FIGURE 7 Nodule formation of *chit5b* and *nfh1-3b* mutant plants inoculated with *S. meliloti* Rm41. *M. truncatula* R108 wild-type (WT) plants were included into the experiment. Plants were harvested at 3 wpi. Data indicate means ± SE (*n* = 10). **(A)** Quantification of the nodule number per plant. **(B)** Quantification of the nodule fresh weight (FW) per plant.

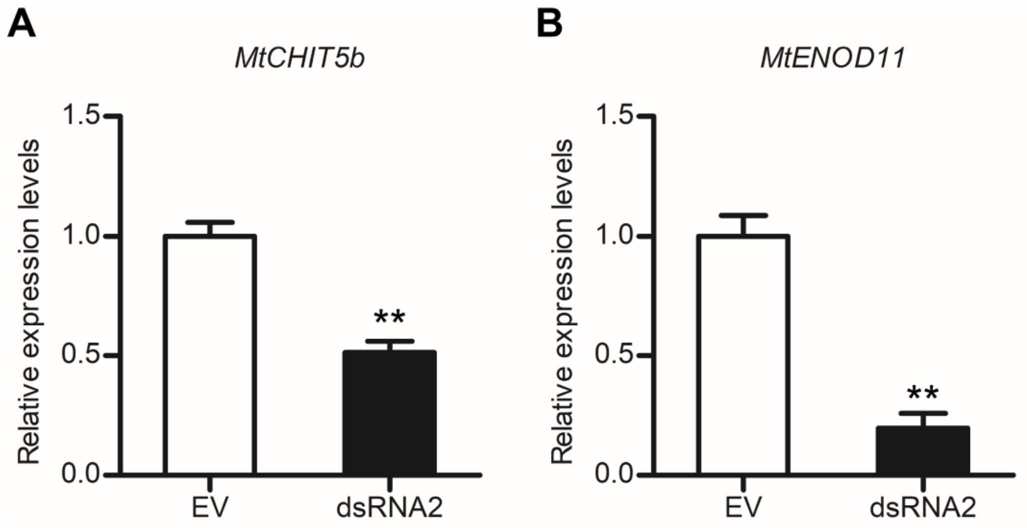

SUPPLEMENTARY FIGURE 8 Expression analysis of *MtCHIT5b* and *MtENOD11* in dsRNA2-transformed *M. truncatula* roots treated with NodSm-IV(C16:2, S). R108 (wild-type) roots were transformed with the dsRNA2 construct or the empty vector (EV). Co-expression of *RFP* allowed identification of transgenic hairy roots at 17 days post *A. rhizogenes*-mediated transformation. Transgenic roots were then treated with liquid BNM containing 0.5% (v/v) DMSO and 10^{-7} M NodSm-IV(C16:2, S). After incubation for 24 h, roots were harvested and used for RT-qPCR analysis. **(A)** *MtCHIT5b* expression **(B)** *MtENOD11* expression. Data indicate means ± SE of normalized values (n = 3; mean expression value of the empty vector control set to one) (Student's t-test; **, $P < 0.01$).

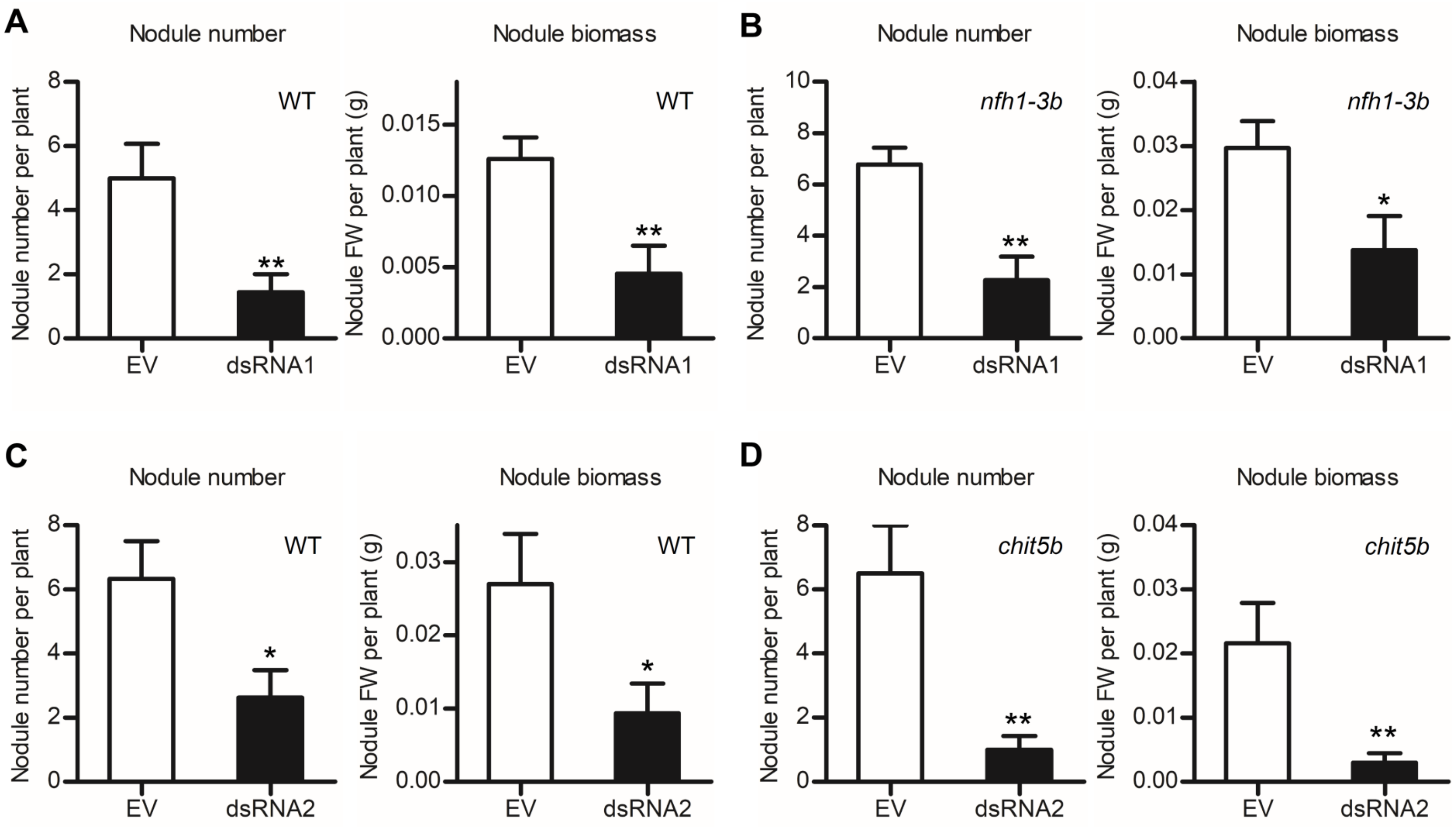

SUPPLEMENTARY FIGURE 9 Nodule parameters on a whole-plant basis obtained from nodulated *M. truncatula* roots transformed with dsRNA1 or dsRNA2 constructs. Roots of R108 wild-type (WT), *chit5b* or *nfh1*-3b plants were transformed with dsRNA1, dsRNA2 or the empty vector (EV). Co-expression of *RFP* allowed identification of transgenic roots. Plants with red fluorescent roots were inoculated with *S. meliloti* Rm41. The number of nodules per plant and the nodule biomass (fresh weight, FW) per plant were determined at the time of harvest (4 wpi). Data indicate means ± SE (Student's *t* test; *, $P < 0.05$; **, $P < 0.01$). **(A)** Nodule parameters of dsRNA1-transformed WT roots (EV, $n = 7$; dsRNA1, $n = 9$)**. (B)** Nodule parameters of dsRNA1-transformed *nfh1-3b* mutant roots (EV, $n = 9$; dsRNA1, $n = 11$). **(C)** Nodule parameters of dsRNA2-transformed WT roots (EV, $n = 9$; dsRNA2, $n = 8$). **(D)** Nodule parameters of dsRNA2-transformed *chit5b* mutant roots (EV, $n = 10$; dsRNA2, $n = 12$).

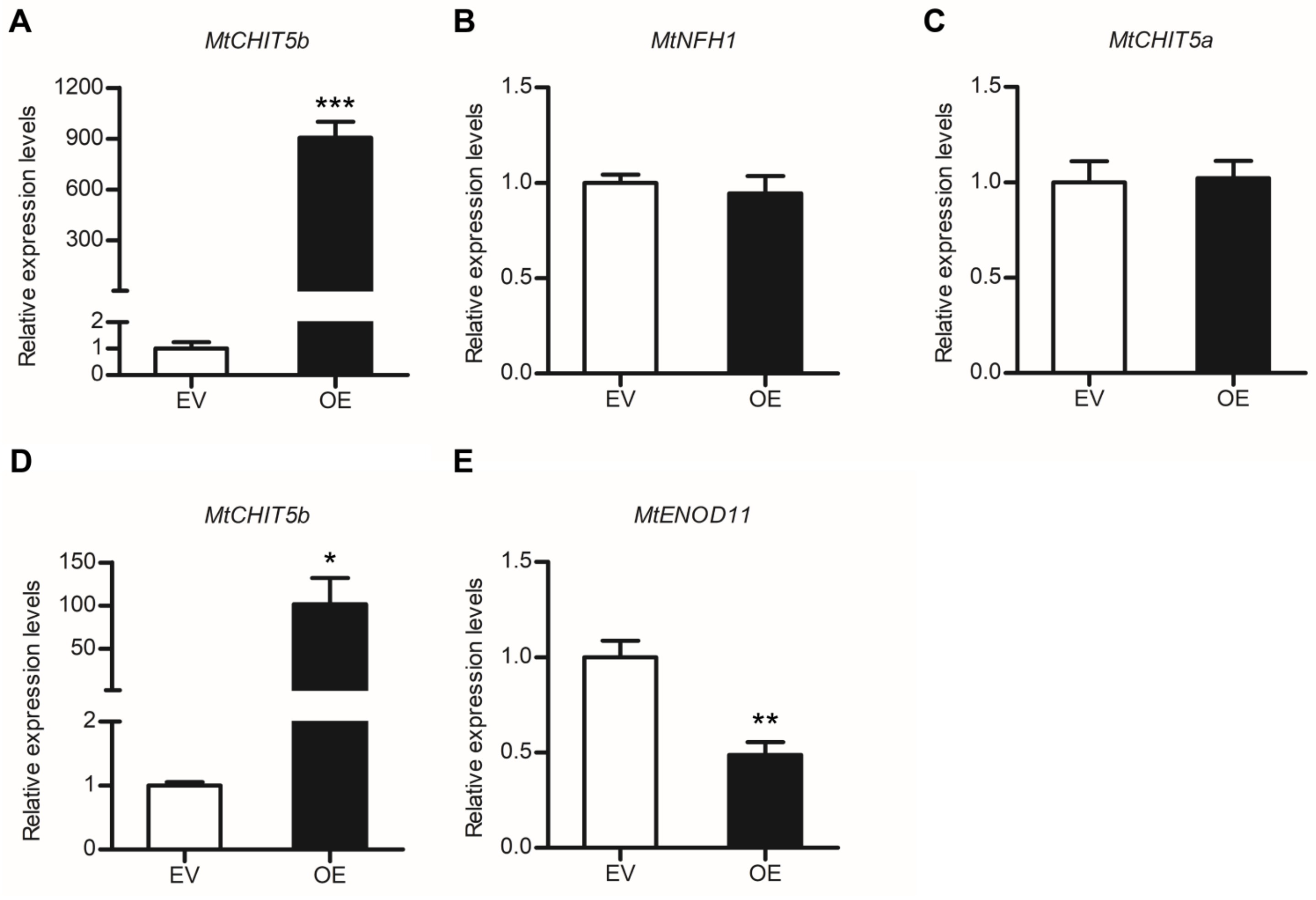

SUPPLEMENTARY FIGURE 10 RT-qPCR analysis of *M. truncatula* roots constitutively overexpressing *MtCHIT5b*.
A. rhizogenes-mediated transformation was used to obtain R108 plants constitutively overexpressing *MtCHIT5b* in roots (OE). Co-expression of *RFP* allowed identification of transgenic roots. Plants transformed with the empty vector containing *RFP* alone (EV) served as a control. **(A-C)** Transgenic roots were directly subjected to RT-qPCR analysis. **(A)** *MtCHIT5b*. **(B)** *MtNFH1*. **(C)** *MtCHIT5a*. **(D-E)** Transgenic roots were treated with liquid BNM containing 0.5% (v/v) DMSO and 10^{-7} M NodSm-IV(C16:2, S). After incubation for 24 h, roots were harvested and used for RT-qPCR analysis. **(D)** *MtCHIT5b*. **(E)** *MtENOD11*. Data indicate means ± SE of normalized values (n = 3; mean expression value of the empty vector control set to one) (Student's *t*-test; *, $P < 0.05$; **, $P < 0.01$; ***, $P < 0.001$).

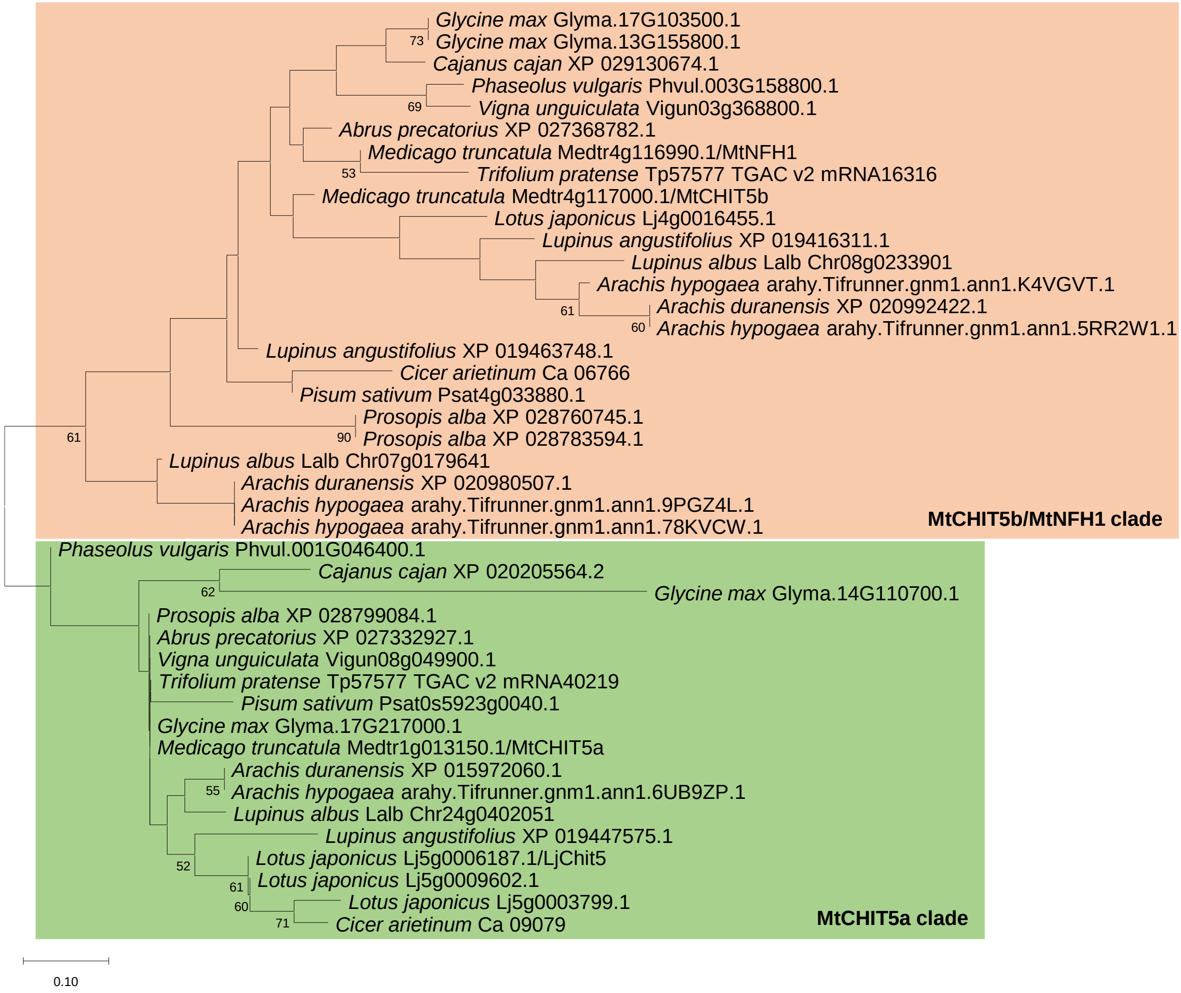

SUPPLEMENTARY FIGURE 11 Phylogenetic analysis of leguminous class V chitinase protein sequences.

Phylogenetic analysis of predicted class V chitinase protein sequences from various legumes was conducted using MEGA11.0 software and the Neighbor-Joining method (Tamura et al., 2021). Bootstrap values greater than 50 (1 000 replicates) are shown at branching points. The protein sequences were obtained by BLAST searches at the NCBI homepage (genome database; https://www.ncbi.nlm.nih.gov/genome/?term=), the Phytozome v13 homepage (https://phytozome-next.jgi.doe.gov/) and the Pulse Crop Database homepage (https://www.pulsedb.org/) using MtCHIT5b, MtNFH1 and MtCHIT5a as query sequences.

Reference: Tamura, K., Stecher, G., and Kumar, S. (2021). MEGA11: Molecular evolutionary genetics analysis version 11. *Mol. Biol. Evol.* 38: 3022–3027.
